# Synthesis and Structure of an S‐Shaped Double Expanded Helicene With Seven Fused Anthracene Units

**DOI:** 10.1002/chem.71249

**Published:** 2026-06-08

**Authors:** Shinji Toyota, Kaito Hohno, Takehiro Kihara, Yu Watanabe, Yuki Shigihara, Eiji Tsurumaki, Masahiro Yamashina

**Affiliations:** ^1^ Department of Chemistry School of Science Institute of Science Tokyo Meguro‐ku Tokyo Japan

**Keywords:** arenes, calculations, cycloisomerization, density functional, double helicene, X‐ray analysis

## Abstract

A long double expanded helicene consisting of seven fused anthracene units was synthesized. After an oligoarene consisting of four ethynyl groups was prepared from a 1,5‐diborylanthracene, four‐fold intramolecular alkyne cycloisomerization of the precursor with PtCl_2_ catalyst yielded the desired compound as an orange solid in 13% yield. X‐ray analysis revealed that this compound has an S‐shaped nonplanar framework of approximately *C_i_* symmetry with two helical sites, *P*,*P*,*P*/*M*,*M*,*M* form, where the two terminal anthracene units are stacked to the central anthracene unit on opposite sides via π–π interactions. DFT calculations indicated the existence of multiple conformers that differ in the helicity of one or two [4]helicene moieties. In the UV/vis and fluorescence spectra, the absorption and emission bands were broad and red‐shifted relative to those of the short double expanded helicenes. The structural and spectroscopic features are discussed on the basis of intramolecular interactions and molecular strain.

## Graphical abstract

The anthracene‐fused aromatic compound was synthesized by four‐fold cycloisomerization of the corresponding alkyne precursor. X‐ray crystallographic analysis revealed that this compound had a double helical structure of *C_i_* symmetry, in which the terminal and central anthracene units were triply stacked. The conformation, intramolecular interactions, and molecular strain were analyzed using theoretical methods.

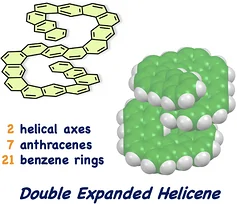

## 1 Introduction

Helicenes consisting of several angularly fused benzene rings are an attractive motif of nonplanar polycyclic hydrocarbons due to their unique structure, chirality, and properties [1, 2, 3, 4]. A large number of helicene derivatives have been synthesized to construct various helical structures and to investigate their optical and chiroptical properties [5, 6, 7, 8]. One structural modification to the original helicene involves the insertion of linearly fused benzene rings to enlarge the helical diameter. These compounds are known as “expanded helicenes” [9, 10], and several types of expanded helicenes have been designed by changing the number and combinations of linearly fused rings [11]. Recently, very long expanded helicenes were reported, some of which were resolved into separable enantiomers, even though they have flexible structures compared with the original helicenes [12, 13, 14].

**FIGURE 1:**
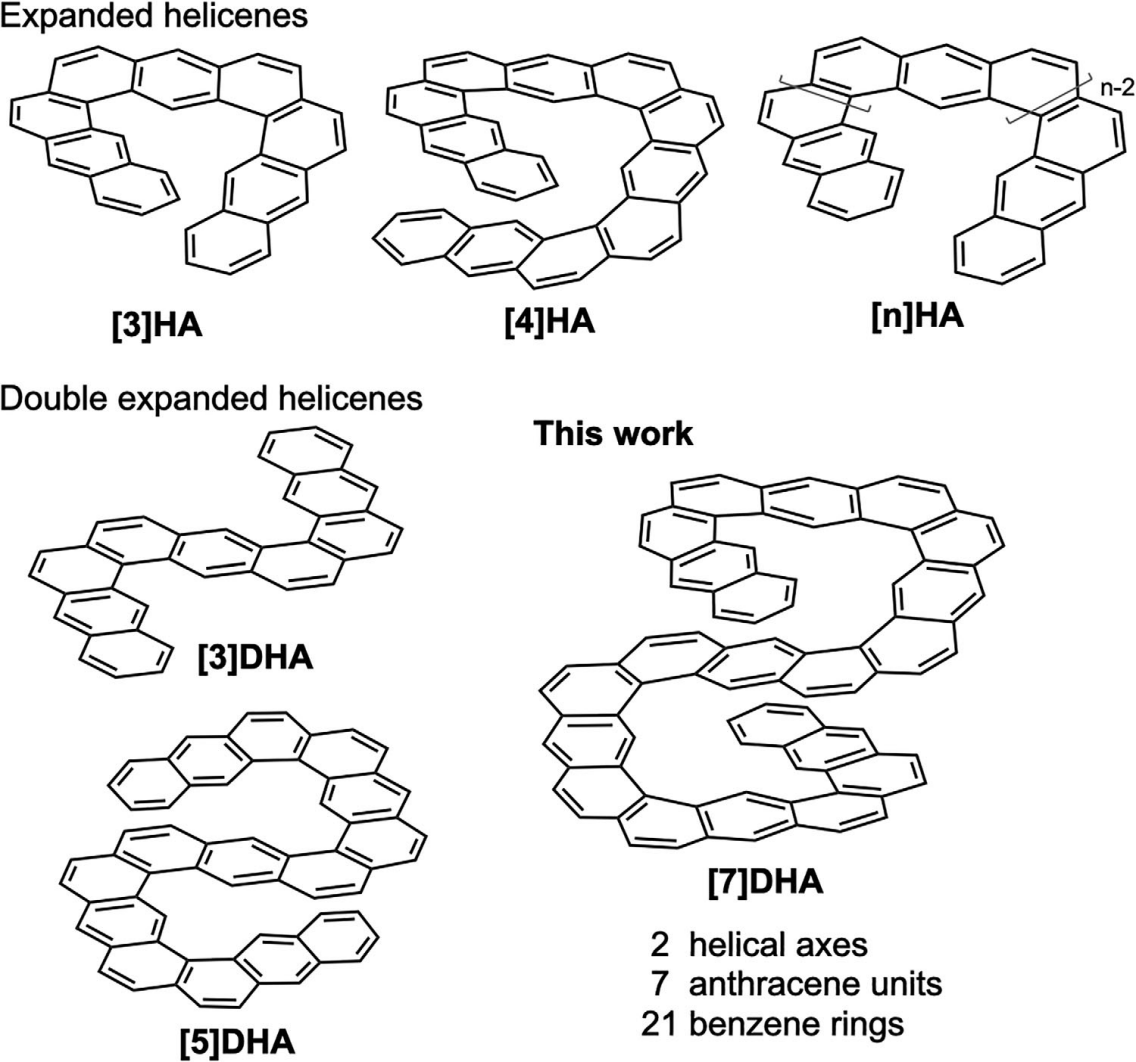
Structures of anthracene‐fused expanded helicenes **[n]HA** and double expanded helicenes **[n]DHA**.

We reported helical fused anthracenes **[n]HA** consisting of n anthracene units fused at 60° angle to form a triangular‐shaped helix (Figure 1) [15, 16, 17, 18]. The longest analog so far synthesized is **[7]HA**, which has seven anthracene units and its helical turn number exceeds two [19]. The enantiomers of **[7]HA** were resolvable but readily racemized at room temperature. Then, we designed expanded helicenes with two helical sites as expanded analog of double helicenes. Multiple helicenes, in particular double helicenes, are attractive targets for the following reasons: structural diversity due to the presence of two helical sites, model compounds of twisted nanographenes, and enhancement of optical and chiroptical properties [20, 21, 22, 23, 24, 25]. As such compounds, we reported the synthesis of two double expanded helicenes, **[3]DHA** and **[5]DHA**, with two helical sites, as well as their structures and dynamic behavior [26, 27]. These compounds have nonplanar S‐shaped structures that readily exchange between some conformers. However, these structures are not long enough to allow the aromatic moieties to stack intramolecularly. Therefore, we designed **[7]DHA** with seven anthracene units with two helical axes. Since this molecule contains two **[4]HA** substructures with a helical turn number exceeding one, the terminal anthracene units interact with other moieties. We herein report the synthesis, structure, and spectroscopic properties of the new long double expanded helicene. The intramolecular interactions and conformational behavior are investigated using theoretical approaches.

## 2 Results and Discussion

### 2.1 Synthesis and Characterization

**SCHEME 1:**
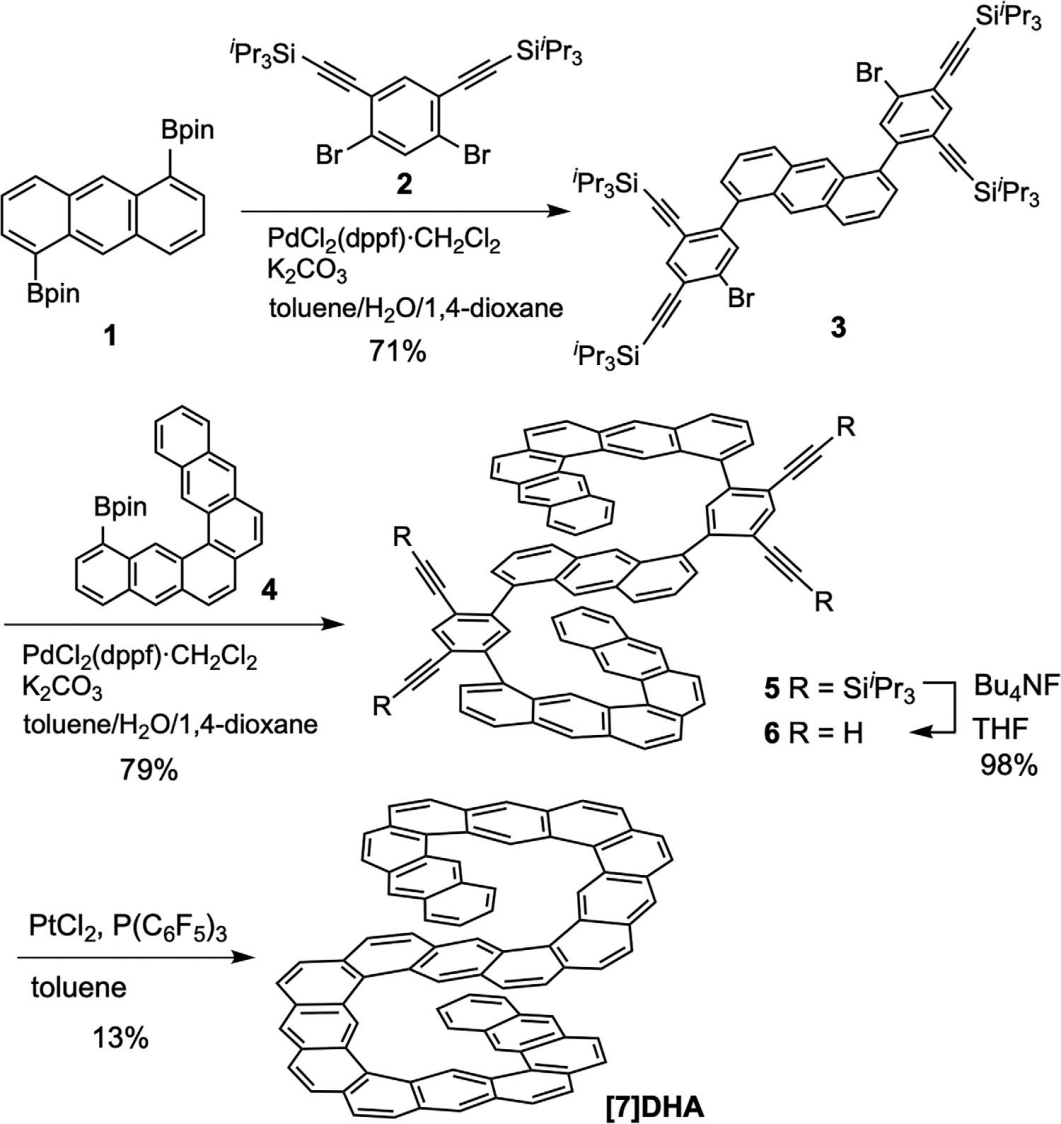
Synthesis of **[7]DHA**.

The target compound **[7]DHA** was synthesized according to Scheme 1. The Suzuki–Miyaura coupling of 1,5‐diborylanthracene **1** [28] with a large amount of *m*‐dibromobenzene **2** [15] afforded compound **3** in 71% yield. Two **[2]HA** units were introduced at the terminal positions by the Suzuki–Miyaura coupling of **3** and **4** to produce oligoarene **5**. After desilylation of **5** with tetrabutylammonium fluoride, the terminal alkyne **6** was heated with PtCl_2_ and P(C_6_F_5_)_3_ in toluene at 110°C for 24 h [29]. The crude products were separated by chromatography on silica gel to afford **[7]DHA** in 13% isolated yield as an orange solid. In its mass spectrum, a molecular ion peak was observed at *m*/*z* 1078.36 (C_86_H_46_).

**FIGURE 2:**
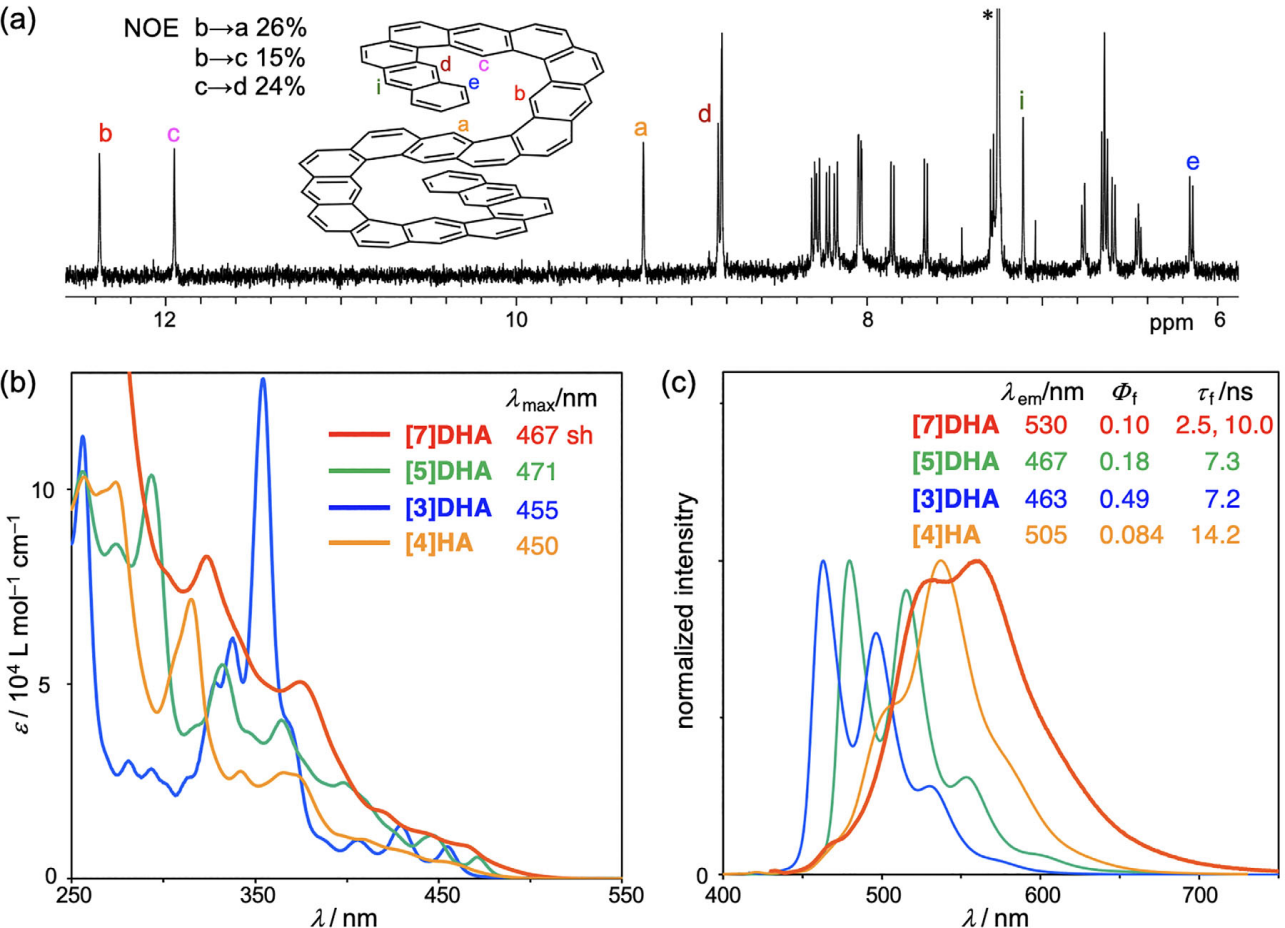
(a) ^1^H NMR spectrum of **[7]DHA** in CDCl_3_ (*: CHCl_3_). (b) UV/vis and (c) fluorescence spectra of **[7]DHA**, **[5]DHA**, **[3]DHA**, and **[4]HA** in CHCl_3_ at 1.0 × 10^−5^ mol L^−1^ (UV/vis) or 1.0 × 10^−6^ mol L^−1^ (FL). sh: shoulder peak.

The ^1^H NMR spectrum of **[7]DHA** exhibited several peaks in the aromatic region (Figure 2a). Two singlets at 12.4 and 12.0 ppm in the downfield region are assigned to the inner H atoms, H_b_ and H_c_, respectively. This deshielding is attributed to the steric compression effect [30, 31] of the sterically congested H atoms, as observed for other **[n]HA** derivatives [15, 16, 19]. Actually, large nuclear Overhauser effects (NOE) were observed between the inner H atoms (Figure S11). In contrast, some signals appeared in the upfield region. For example, a doublet at 6.1 ppm was assigned to the H_e_ atoms in the terminal benzo groups. These protons are shielded by the ring current effect of the adjacent aromatic moiety.

### 2.2 UV/vis and Fluorescence Spectra

The UV/vis and fluorescence spectra of **[7]DHA** measured in CHCl_3_ are compared with those of related compounds. In the UV/vis spectrum, **[7]DHA** exhibited broad absorption bands with a shoulder at ca. 470 nm and an edge extending to 500 nm (Figure 2b). The absorption edges red‐shifted and broadened in the order of **[3]DHA**, **[5]DHA**, and **[7]DHA**. In the fluorescence spectrum, **[7]DHA** exhibited broad bands that peaked at 530 and 561 nm, which were significantly broad and red‐shifted compared with those of **[3]DHA** and **[5]DHA** (Figure 2c). Its fluorescence quantum yield (*Φ*_f_ 0.10) is smaller than those of the other compounds: **[3]DHA** (0.49) and **[5]DHA** (0.18). The fluorescence band of **[7]HA** consists of two components decayed at the lifetimes of 2.5 (34%) and 10.0 (66%) ns. The lifetime of the latter major component is longer than those of **[3]DHA** and **[5]DHA**. These spectral features of **[7]DHA** are similar to those of **[4]HA** [16]. Because the aromatic moieties overlap effectively from **[7]DHA** in the double helicene system and from **[4]HA** in the single helicene system, intramolecular π–π stacking should affect their optical properties. In particular, the broad and long‐lived emission is attributed to excimer‐type interactions in the excited state [32, 33, 34].

### 2.3 X‐Ray Crystallographic Analysis

**FIGURE 3:**
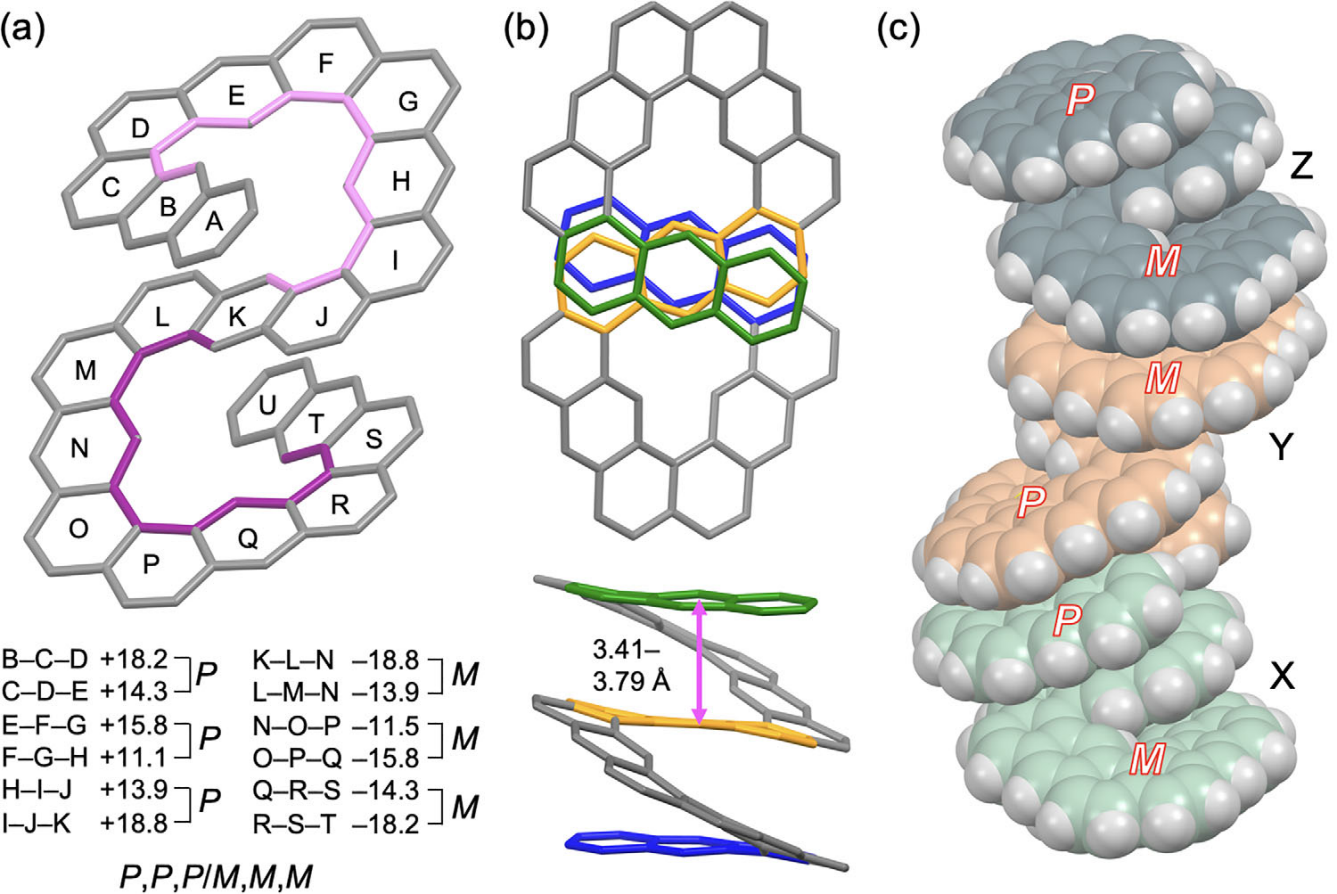
X‐ray structures of **[7]DHA**. Hydrogen atoms and solvent molecules are omitted. (a) Stick model with torsion angles (°) along the inner carbon rims shown in violet and purple colors. (b) Two other stick models with highlights of the terminal two anthracene units (green and blue) and the central anthracene (orange). (c) Part of stacking array along the *c* axis as space‐filling model in the packing diagram (see also Figure S1).

We performed X‐ray crystallographic analysis of **[7]DHA** with a single crystal prepared from a CH_2_Cl_2_/hexane solution [35]. The structure shown in Figure 3 is an S‐shaped nonplanar aromatic framework of *C_i_* symmetry, where the two helical sites have *P* and *M* global helicity. The central anthracene unit is sandwiched by the terminal anthracene units (*anti*), and the separations of the triple stacking at 3.4–3.8 Å suggest the presence of π–π interactions (Figure 3b). This molecule has two **[4]HA** substructures, each of which contains three [4]helicene substructures. The dihedral angles along the inner rim indicate that the local helicity of the three [4]helicene substructures in one **[4]HA** moiety is *P*,*P*,*P* and that in another **[4]HA** moiety is *M*,*M*,*M* (Figure 3a). Hereinafter, this local stereochemistry is designated as *P*,*P*,*P*/*M*,*M*,*M* with a slash separating the two helical moieties. Accordingly, the molecule extends in both directions along the helical axes like a double spring. This structure explains the upfield shift of aromatic signals due to the H atoms in the triply stacking region (e.g., H_e_) in the ^1^H NMR spectrum, resulting from the ring current effect of adjacent aromatic moieties. In the crystal packing, twisted molecules form linear arrays along the *c* axis via effective intermolecular π–π stacking at ca. 3.5–3.8 Å (Figures 3c and S1). To fit the helical shape, a *P* helical moiety in molecule X stacks with a *P* helical moiety in molecule Y, and an *M* helical moiety in Y stacks with an *M* helical moiety in the next molecule Z, forming a successive *M*–*P*···*P*–*M*···*M*–*P* array.

### 2.4 DFT Calculations

**FIGURE 4:**
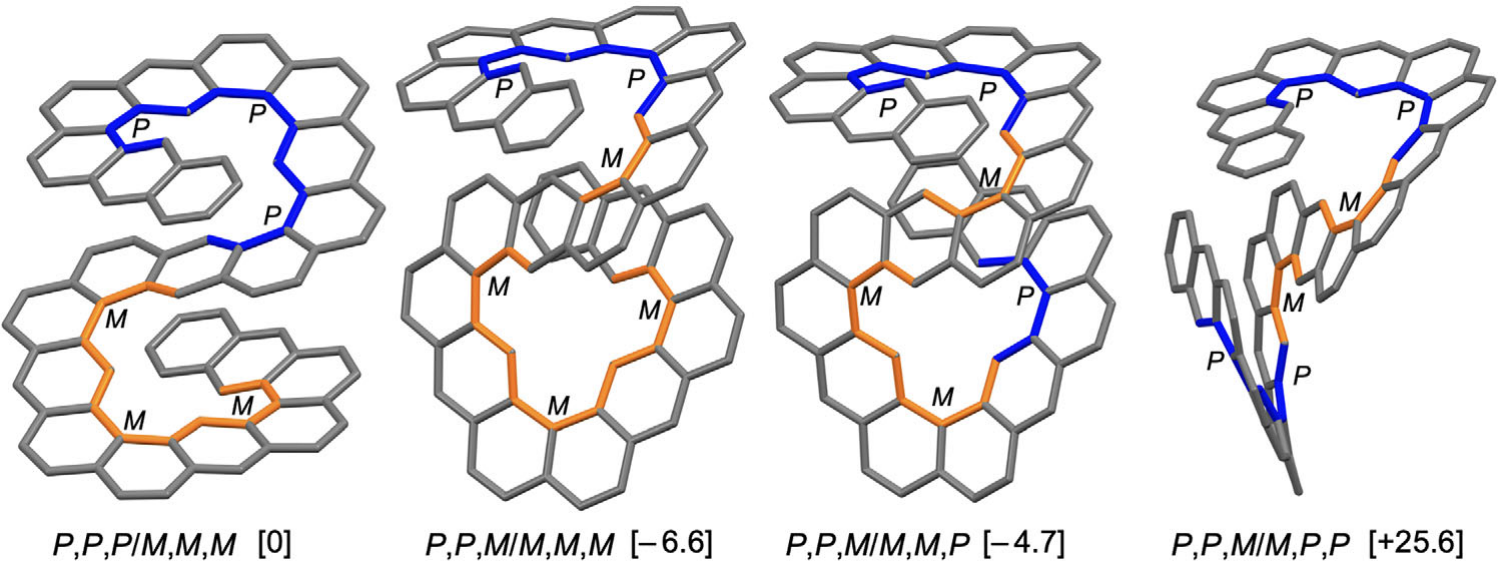
Energy‐minimum structures of **[7]DHA** calculated at the B3LYP‐D3/6‐31G(d,p) level. Relative Gibbs free energies Δ*G*_298_ are given in brackets in kJ mol^−1^. Inner rims of [4]helicene moieties of *M* and *P* helicity are highlighted in orange and blue, respectively. Additional structural and energy data are listed in Tables S4 and S5.

We calculated the structure of **[7]DHA** using by the density functional theory (DFT) method at the B3LYP‐D3/6‐31G(d,p) level. Structural optimization gave the *P*,*P*,*P*/*M*,*M*,*M* form similar to the X‐ray structure as an energy‐minimum structure as shown in Figure 4. Further conformational analysis afforded the *P*,*P*,*M*/*M*,*M*,*M* and *P*,*P*,*M*/*M*,*M*,*P* forms, which have comparable energies to the *P*,*P*,*P*/*M*,*M*,*M* form and also belong to the *anti* form. We also found the *P*,*P*,*M*/*M*,*P*,*P* form as a sole energy‐minimum structure with the terminal anthracene units on the same side of the central unit (*syn* form), which was much less stable than the other forms. Therefore, **[7]DHA** strongly prefers to adopt one of the *anti* forms to avoid steric hindrance between the terminal anthracene units in the *syn* form. This structural feature contrasts with **[3]DHA** and **[5]DHA**, which can adopt both the *anti* and *syn* forms [26]. Unfortunately, we failed to optimize the transition states of the inversion processes. We adopted the semi‐empirical tight‐binding method (GFN2‐xTB) for the transition state search [36]. The calculations revealed that the barriers to inversion processes (*P*,*P*,*P*/*M*,*M*,*M* → *P*,*P*,*M*/*M*,*M*,*M* → *P*,*P*,*M*/*M*,*M*,*P*), where each step involves the inversion of one helical moiety, were less than 10 kJ mol^−1^ (Figure S5). These low barriers are consistent with that of a similar process in **[4]HA** (ca. 13 kJ mol^−1^ for *P*,*P*,*P* → *P*,*P*,*M*) [16]. Therefore, the three *anti* conformers should interconvert very rapidly even at low temperatures, resulting in only one set of signals in the ^1^H NMR spectra. We could not calculate the transition state of the *P*,*P*,*M*/*M*,*M*,*P* → *P*,*P*,*M*/*M*,*P*,*P* process because the transition state was considerably strained.

**FIGURE 5:**
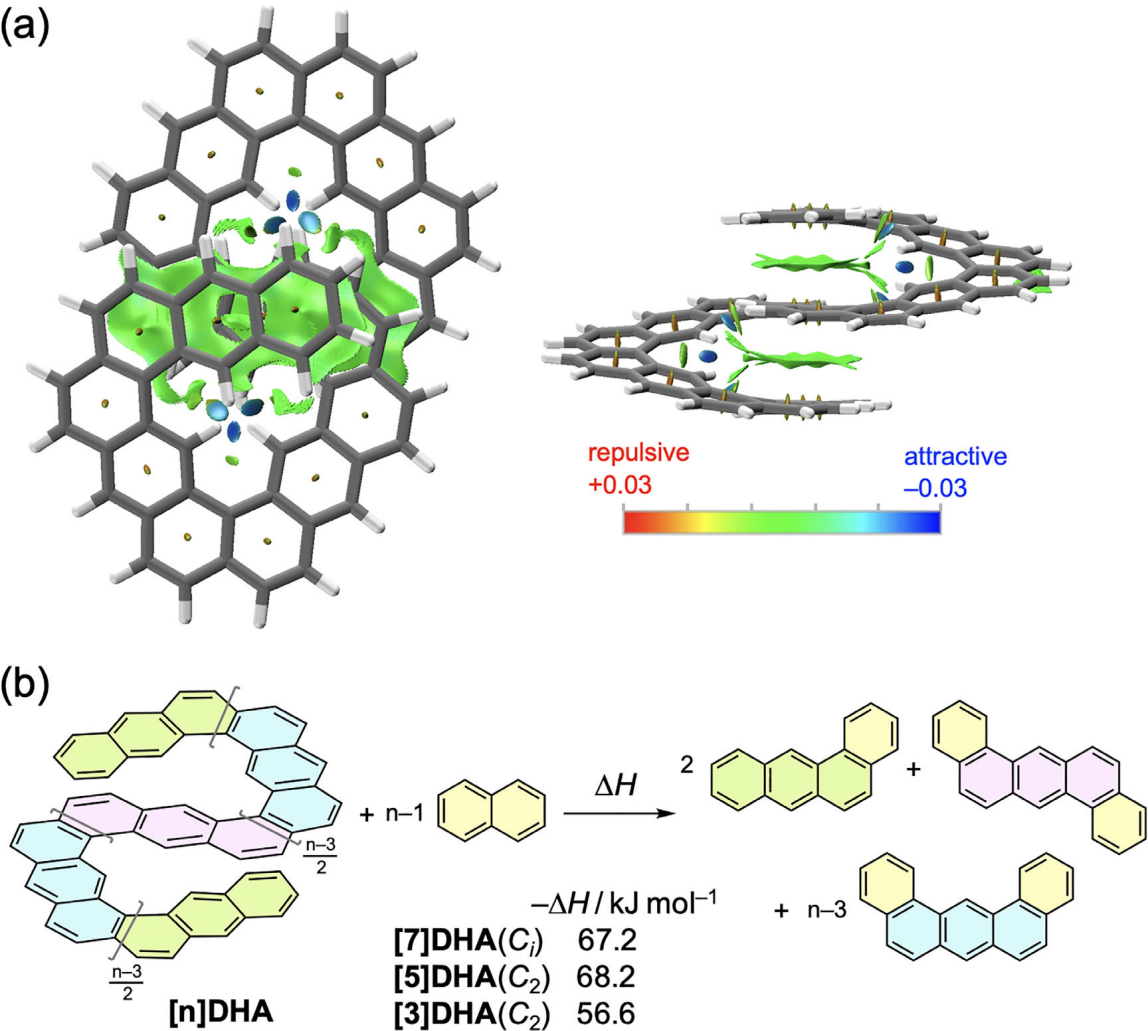
(a) Two views of NCI plot of **[7]DHA**. Scale –0.03 a.u. (blue, attractive) ≤ sign(*λ*_2_)*ρ* ≤ +0.03 a.u. (red, repulsive) and isosurface 0.50 a.u. (b) Homodesmotic reactions and enthalpies of reaction for **[n]DHA** calculated at the B3LYP‐D3/6‐31G(d,p) level. The enthalpy of the stable conformer was adopted for each **[n]DHA**.

Intramolecular interactions in the calculated *P*,*P*,*P*/*M*,*M*,*M* form were visualized by the noncovalent interaction (NCI) plot as shown in Figure 5a [37]. Wide green isosurfaces spread between the terminal and central anthracene units suggest triple‐layered π–π interactions. Isosurfaces in the inner regions indicate nonbonded H–H interactions with interatomic distances (1.75 and 1.63 Å) significantly smaller than the sum of the van der Waals radii of the H atom (2.4 Å). These interactions are the main reason for the deshielding of these atoms in the ^1^H NMR spectrum. To access the molecular strain and interactions, we performed a thermochemical analysis using the homodesmotic reaction shown in Figure 5b [38, 39]. Negative values of the enthalpy of reaction (–Δ*H*) indicate the stability of the double helicene in the reactant system. The values are 56.6, 68.2, and 66.6 kJ mol^−1^ for **[3]DHA**, **[5]DHA**, and **[7]DHA**, respectively. If out‐of‐plane helical deformation were a major factor in stability, the –Δ*H* values would increase additively with increasing the number of anthracene units. These results mean that the additional strain in **[7]DHA** is nearly canceled by attractive π–π interactions. This tendency was also observed in the **[n]HA** system, where the stabilization by π–π interactions became significant for the single expanded helicenes (*n* ≥ 4) with turn numbers exceeding one [16, 19]. Nucleus‐independent chemical shifts (NICS) values were calculated for each benzene ring in **[7]DHA** at the GIAO‐B3LYP‐D3/6‐31G(d,p) level (Table S2) [40, 41]. The aromaticity is high for the linearly fused and terminal benzene rings and low for the angularly fused benzene rings as with general all‐*ortho* fused aromatic compounds [42, 43].

**FIGURE 6:**
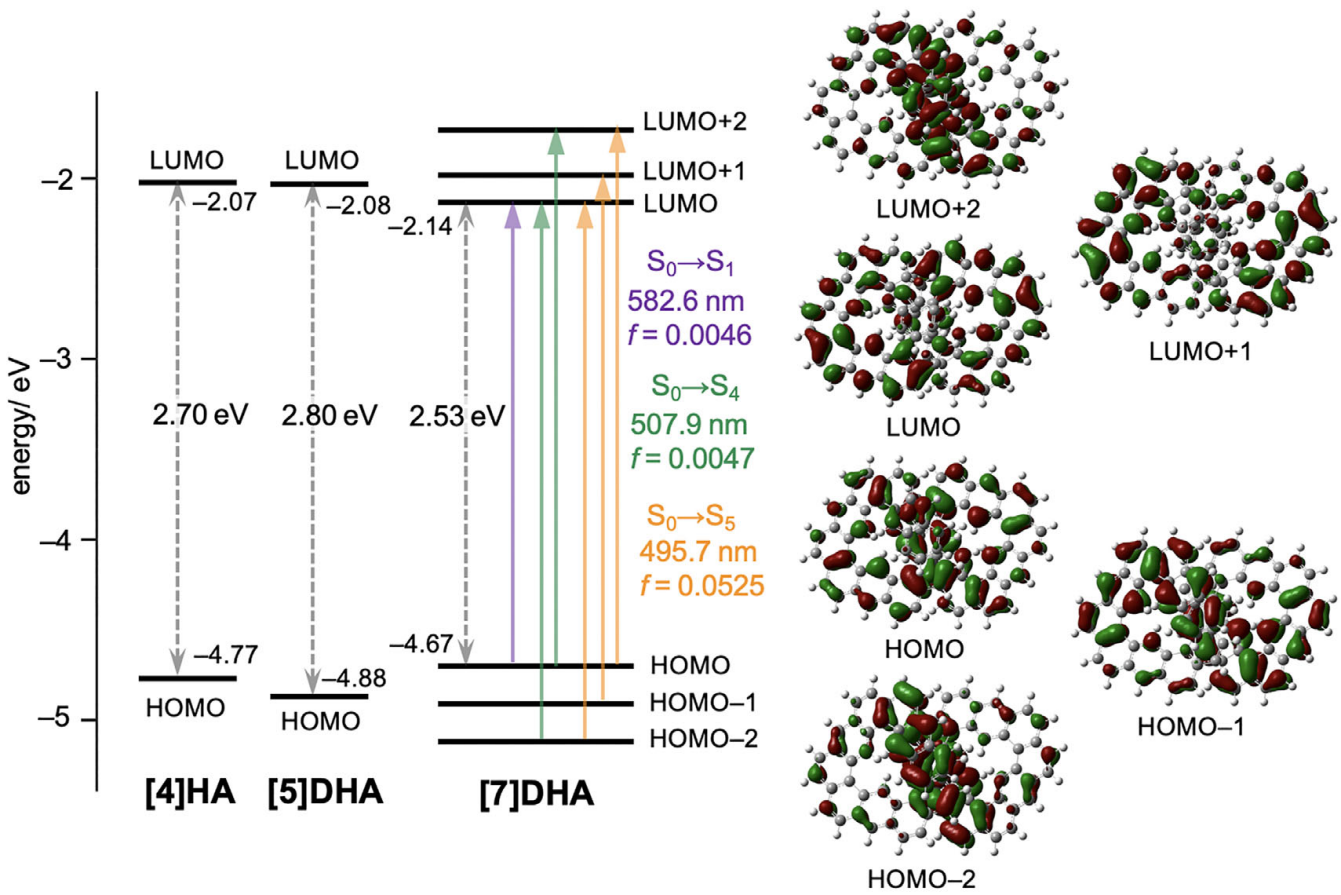
Energy diagrams and orbital distributions of frontier orbitals of **[7]DHA** (*C_i_*) and its related compounds calculated at the B3LYP‐D3/6‐31G(d,p) level. HOMO–LUMO gaps and selected TD‐DFT data are also included.

The HOMO and LUMO diagrams and their energies are shown in Figure 6. The orbital lobes spread over the molecule at the HOMO and LUMO levels. The HOMO level of **[7]DHA** (*C_i_*) is elevated compared with those of the other compounds. As a result, the HOMO–LUMO gap of **[7]DHA** (2.53 eV) is narrower than those of **[5]DHA** (2.80 eV) and **[4]HA** (2.70 eV). The time‐dependent (TD)‐DFT calculation revealed that the S_0_ → S_1_ transition due to the HOMO → LUMO excitation is very weak, and several other transitions are forbidden due to the symmetric structure. The first intense absorption is attributed to S_0_ → S_5_ transition at 496 nm (*f* = 0.0525), which corresponds to the broad absorption in this region of the observed spectrum (Figure S4 and Table S1). The broadening is also attributed to the presence of multiple conformers as discussed above.

## 3 Conclusions

In conclusion, we successfully synthesized the double expanded helicene with seven fused anthracene units by alkyne cycloisomerization. X‐ray structure revealed that the molecules take an S‐shaped structure of *C_i_* symmetry, where the central anthracene is stacked with two terminal anthracene units. Intramolecular π–π interactions in the triply stacked aromatic units and nonbonded H–H interactions significantly influence the molecular structure and spectroscopic properties. Conformational analysis suggests that the molecules can take multiple conformations within the *anti* form. To create diverse helical structures, further studies are in progress to synthesize other types of double and multiple expanded helicenes, which could be key substructures of twisted nanographenes. Chiral double helicenes are an attractive target for generating chiroptical activity in the fused anthracene system.

## 4 Experimental Section

The synthetic procedures of the final step and the physical and spectroscopic data of the final product are described here. Other materials are provided in Supporting Information.

### 4.1 Synthesis of **[7]DHA**

A solution of **6** (38.5 mg, 35.7 µmol), PtCl_2_ (4.1 mg, 15 µmol), and P(C_6_F_5_)_3_ (7.2 mg, 14 µmol) in anhydrous toluene (6.4 mL) was stirred for 24 h at 110°C under N_2_ atmosphere. The resultant mixture was evaporated under reduced pressure. The crude product was purified by column chromatography on silica gel using hexane/CH_2_Cl_2_/CS_2_ (1:1:2) as eluent. The residue was washed with MeOH to give the desired product as an orange solid. Yield 4.89 mg (13%); mp 325–329°C (dec); *R*_f_ = 0.32 (hexane/CH_2_Cl_2_ 2:1); ^1^H NMR (500 MHz, CDCl_3_): *δ* = 6.16 (d, *J* = 8.0 Hz, 2H), 6.46 (t, *J* = 7.3 Hz, 2H), 6.60 (d, *J* = 8.5 Hz, 2H), 6.63–6.68 (m, 6H), 6.78 (d, *J* = 8.5 Hz, 2H), 7.12 (s, 2H), 7.30 (d, *J* = 8.5 Hz, 2H), 7.67 (d, *J* = 8.5 Hz, 2H), 7.86 (d, *J* = 8.0 Hz, 2H), 8.047 (d, *J* = 8.5 Hz, 2H), 8.051 (d, *J* = 9.0 Hz, 2H), 8.19 (d, *J* = 9.0 Hz, 2H), 8.23 (d, *J* = 8.5 Hz, 2H), 8.29 (d, *J* = 8.5 Hz, 2H), 8.31 (d, *J* = 8.5 Hz, 2H), 8.83–8.86 (m, 6H), 9.28 (s, 2H), 11.95 (s, 2H), 12.38 (s, 2H); ^13^C{^1^H} NMR (151 MHz, CDCl_3_/CS_2_ 9:1): *δ* = 124.59, 125.06, 125.29, 125.36, 125.58, 125.62, 125.66, 126.01, 126.12, 126.23,126.42, 126.99, 127.19, 127.27, 127.33, 127.51, 127.61, 127.65, 127.74, 127.78, 127.86, 127.92, 127.96, 128.20, 128.61, 129.49, 129.54, 129.58, 129.83, 130.35, 130.64, 130.70, 130.74, 130.80, 130.86, 131.17, 131.24, 131.29, 131.31, 131.51, 132.07 (two signals were overlapped); HRMS (MALDI‐TOF): *m*/*z* calc. for C_86_H_46_ 1078.3594 [M]^+^; found: 1078.3616; UV/vis (CHCl_3_, 1.0 × 10^−5^ mol L^−1^): *λ*_max_ (*ε*) = 257 (158,000), 273 (154,000), 323 (82,800), 374 (50,600), 421 (16,900, sh), 446 (11,200, sh), 467 nm (8,110 L mol^−1^ cm^−1^, sh); FL (CHCl_3_, 1.0 × 10^−6^ mol L^−1^): *λ*_em_ = 530, 561 nm, *λ*_ex_ = 420 nm (*Φ*_f_ = 0.10), *τ*_f_ = 9.2 ns (2.5 ns (34%) and 10.0 ns (66%)) at 530 nm.

## Conflicts of Interest

The authors declare no conflicts of interest.

## Supporting information




**Supporting file**: Experimental details, X‐ray crystallographic analysis, computational methods, NMR charts, and coordinates of the calculated structure. The authors have cited additional references within the Supporting Information [44, 45, 46, 47, 48, 49, 50].

**Supporting file**: chem71249‐sup‐0002‐cif.zip.

## Data Availability

The data that support the findings of this study are available from the corresponding author upon reasonable request.
