# Supplementary material for: Synthesis and Structure of an S‐Shaped Double Expanded Helicene With Seven Fused Anthracene Units
Source: Chemistry. 2026 Jun 8;32(35):e71249. doi: 10.1002/chem.71249 (PMC13587644; doi:10.1002/chem.71249)
Supplement: Supplementary file 1 [file CHEM-32-e71249-s001.pdf]

Supporting Information

1. Experimental details S1

2. X-ray crystallographic analysis S4

3. Computational methods S6

4. References S12

5. NMR charts S13

6. Coordinates of calculated structures S19

1. Experimental details

General

Melting points were measured with a Yanako Technical Science MP-500P and are uncorrected. NMR spectra were measured on a JEOL JNM-ECX500 spectrometer (^{1}H: 500 MHz, ^{13}C: 125 MHz) or a Bruker 600 MHz Avance III HD Smart Probe NMR spectrometer (^{1}H: 600 MHz, ^{13}C: 151 MHz). High-resolution MALDI-TOF mass spectra were measured on a Bruker UltrafleXtreme mass spectrometer using DCTB (*trans*-2-[3-(4-*t*-butylphenyl)-2-methyl-2-propenylidene]malononitrile) as a matrix and PEG-1000 or PEG-2000 (polyethylene glycol) as a calibrant. UV/Vis spectra were measured on a JASCO V-730 spectrometer with a 10 mm cell. Fluorescence spectra were measured on a JASCO FP-8550 spectrofluorometer with a 10 mm cell. Absolute fluorescence quantum yields were recorded on the JASCO FP-8550 spectrofluorometer with a JASCO ILF-135 120 mm integrating sphere with a 10 mm cell. Fluorescence lifetimes were recorded on a Hamamatsu C11367-02 system (λ_{ex} = 365 nm) with a sample degassed immediately prior to measurement.

1,5-Bis(3,3,4,4-tetramethyl-1,3,2-dioxaborolan-1-yl)anthracene (1)

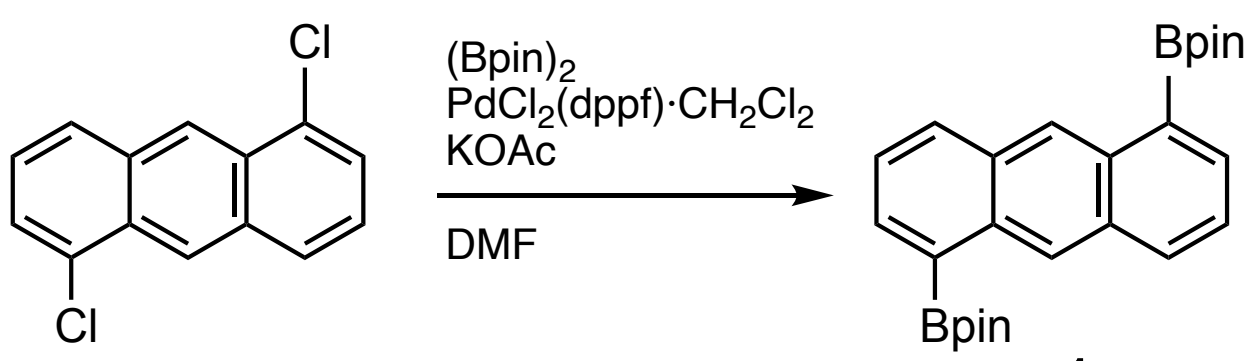

1

This known compound was prepared by the Miyaura-Ishiyama coupling of 1,5-dibromoanthracene in the literature.[S1] This compound was similarly prepared from 1,5-dichloroanthracene as described below. A solution of 1,5-dichloroanthracene (1.01 g, 5.10 mmol), bis(pinacolato)diboron (4.43 g, 8.25 mmol), $PdCl_2$(dppf)·CH_2Cl_2 (0.171 g, 0.209 mmol), and KOAc (2.01 g, 20.5 mmol) in anhydrous DMF (20 mL) was stirred at 140 °C for 24 h under N_2 atmosphere. After cooling to room temperature, the reaction was quenched with H_2O (80 mL). The crude product was filtered through a short pad of silica gel using CH_2Cl_2 as eluent. The residue was washed with MeOH to give the desired product as a pale-yellow solid. Yield 1.37 g (78%); ^{1}H NMR (500 MHz, $CDCl_3$): δ = 9.31 (s, 2H), 8.18 (d, J = 8.6 Hz, 2H), 8.09 (d, J = 6.6 Hz, 2H), 7.45 (m, 2H), 1.47 (s, 24H); ^{13}C{^{1}H} NMR (125 MHz, $CDCl_3$): δ = 136.1, 134.0, 133.0, 132.1, 127.9, 124.5, 83.9, 76.9, 25.2.

1,5-Bis{5-bromo-2,4-bis[(triisopropylsilyl)ethynyl]phenyl}anthracene (3)

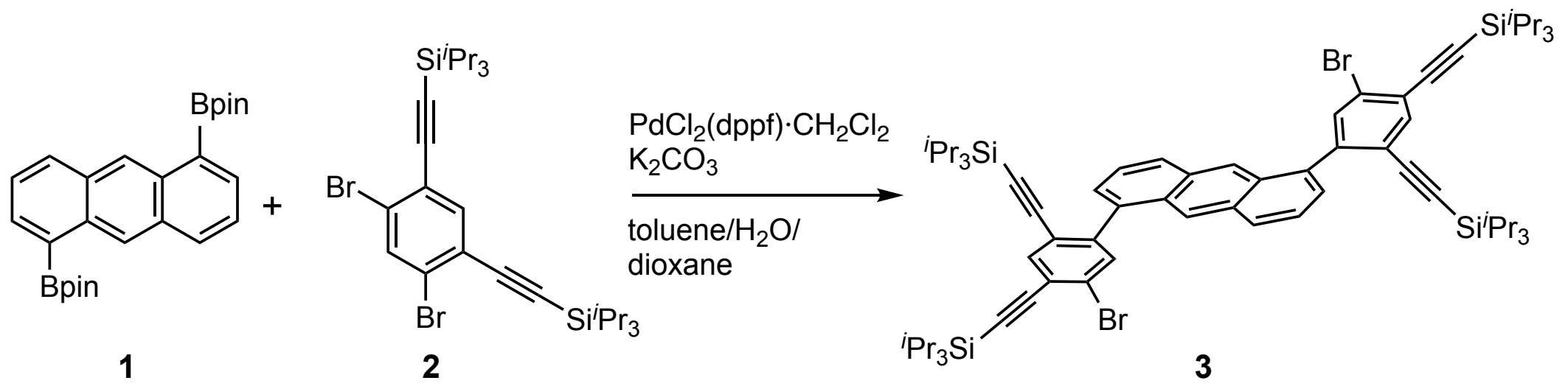

A solution of **1** (0.303 g, 0.705 mmol), **2** [S2] (2.51 g, 4.20 mmol), $PdCl_2(dppf)·CH_2Cl_2$ (32.1 mg, 0.0393 mmol), and K_2CO_3 (311 mg, 2.25 mmol) in a degassed mixture of toluene (5.0 mL), 1,4-dioxane (2.5 mL), and H_2O (2.5 mL) was stirred at 80 ℃ for 19 h under N_2 atmosphere. After cooling to room temperature, the reaction mixture was quenched with H_2O (10 mL). The organic materials were extracted with CH_2Cl_2 (15 mL × 3). The combined organic layer was washed with brine (10 mL), dried over $MgSO_4$, and evaporated. The crude product was purified by column chromatography on silica gel using hexane as eluent. The residue was washed with MeOH to give the desired product as a white solid. The NMR spectra showed the presence of rotamers in ca. 3:1 ratio. Yield 501 mg (71%); mp 342–346 °C (dec.); R_f = 0.24 (hexane); ^{1}H NMR (500 MHz, $CDCl_3$): δ = (major rotamer, readable signals only) 8.08 (s, 2H), 7.86 (d, J = 8.3 Hz, 2H), 7.79 (s, 2H), 7.65 (s, 2H), 7.33 (d, J = 6.6 Hz, 2H); (minor rotamers, readable signals only) 8.17 (s, 2H), 8.89 (d, J = 8.0 Hz, 2H), 7.82 (s, 2H), 7.68 (s, 2H); (signals for the two rotamers) 7.45–7.38 (m), 1.24–1.18 (m), 0.76–0.70 (m), 0.61–0.50 (m); ^{13}C{^{1}H} NMR (125 MHz, $CDCl_3$): δ = (signals for the two rotamers) 145.0, 144.2, 137.9, 137.3, 137.0, 136.4, 134.8, 134.4, 132.2, 132.0, 130.0, 129.9, 129.5, 129.3, 127.5, 127.2, 126.6, 125.7, 125.4, 125.0, 124.7, 124.6, 123.6, 123.3, 104.1, 104.0, 103.9, 97.4, 96.5, 96.2, 18.9, 18.4, 18.3, 11.7, 11.5, 11.3, 11.0, 10.9; HRMS (MALDI-TOF): m/z calc. for $C_{70}H_{96}{}^{79}Br_2Si_4$ 1206.4950 $[M]^+$; found: 1206.4930.

Compound 5

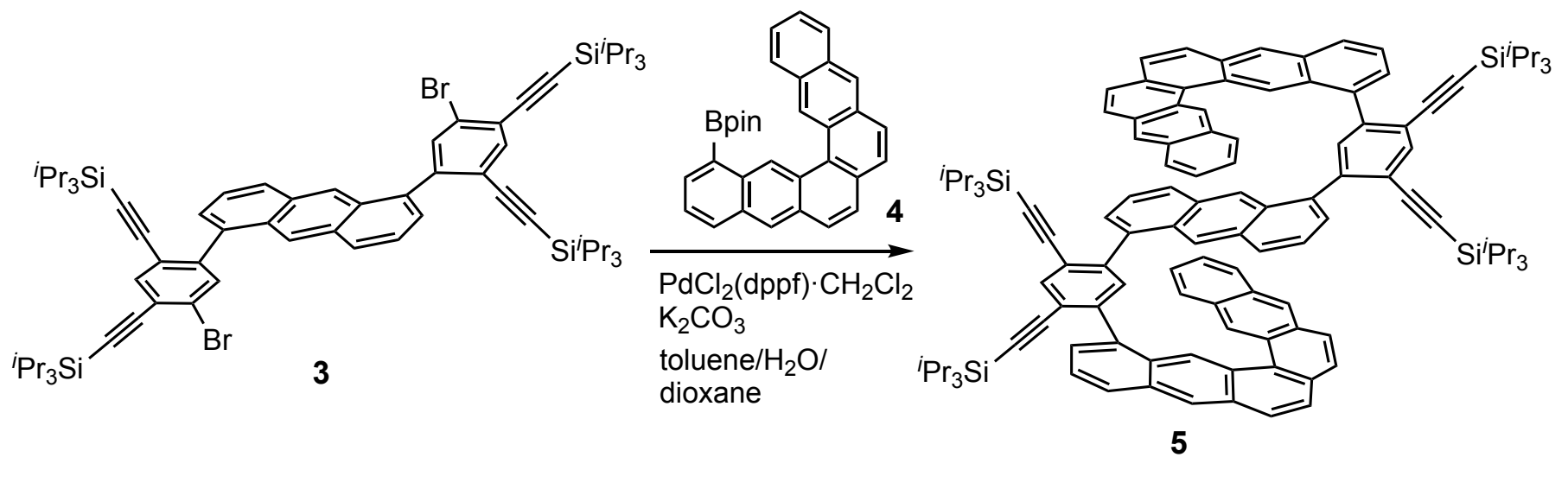

A solution of **3** (0.300 g, 0.248 mmol), 1-(4,4,5,5-tetramethyl-1,3,2-dioxaborolan-2-yl)anthra[1,2-*a*]anthracene (**4**) [S3] (0.237 g 0.520 mmol), and $PdCl_2(dppf)·CH_2Cl_2$ (26.1 mg, 0.0312 mmol), and K_2CO_3 (103 mg, 0.745 mmol) in a degassed mixture of toluene (6.0 mL), 1,4-dioxane (3.0 mL), and H_2O (3.0 mL) was stirred at 100 °C for 22 h under N_2 atmosphere. After cooling to room temperature, the reaction mixture was quenched with H_2O (20 mL). The organic materials were extracted with CH_2Cl_2 (20 mL × 3). The combined organic layer was washed with brine (20 mL), dried over $MgSO_4$, and evaporated. The crude product was purified by column chromatography on silica gel using hexane/CH_2Cl_2 (20:1) as eluent. The residue was washed with MeOH to give the desired product as a yellow solid. Yield 0.334 g (79%); mp 280–285 °C (dec.); R_f = 0.55 (hexane/CH_2Cl_2 4:1); ^{1}H NMR (500

MHz, $CDCl_3$): δ = 0.24–0.83 (several alkyl signals); 6.00–6.20, 6.74, 6.84, 6.94, 7.00, 7.10, 7.15–8.60 (several complicated aromatic peaks); 9.64, 9.71, 9.74, 9.77, 9.79, 9.83–9.94 (several complicated aromatic signals); $^{13}C\{^1H\}$ NMR (125 MHz, $CDCl_3$): δ = 10.6–11.3 (several alkyl signals); 18.0–18.6 (several alkyl signals); 94.8–95.4, 96.2–97.0, 104.5–105.7, 123.2–133.4, 136.5–139.4, 143.2–144.2 (several aromatic signals); HRMS (MALDI-TOF):*m*/*z* calc. for $C_{122}H_{126}Si_4$ 1702.8931 $[M]^+$; found: 1702.8890. The NMR spectra of this compound were very complicated because of the presence of several diastereomeric rotamers in different ratios, so most signals were not readable separately (Figure S7).

Compound 6

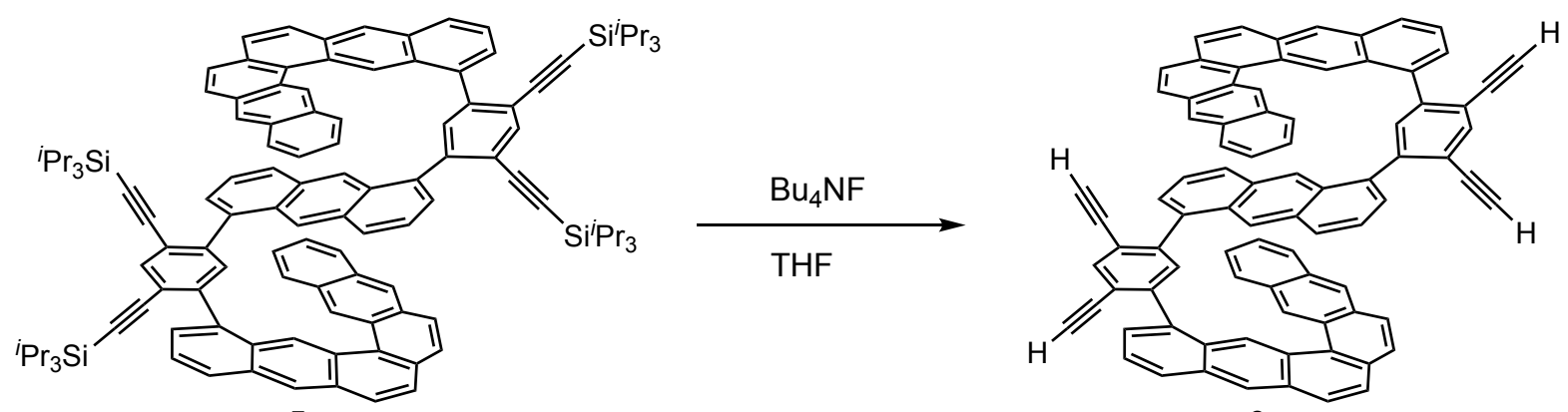

To a solution of **5** (60.5 mg, 35.5 μmol) in THF (4.8 mL) was added the Bu_4NF solution (1.0 mol L^{-1} in THF; 0.156 mL, 0.156 mmol) dropwise. After this mixture was stirred for 1 h at room temperature, the reaction was quenched with H_2O (10 mL). The organic materials were extracted with CH_2Cl_2 (10 mL × 6). The combined organic layer was washed with brine (20 mL), dried over $MgSO_4$, and evaporated. The residue was washed with MeOH to give the desired product as a yellow solid. An analytical sample was purified by chromatography on silica gel using hexane/CH_2Cl_2/CS_2 (3:1:4) as eluent. Yield 36.9 mg (98%); mp 314–317 °C (dec.); R_f = 0.41 (hexane/CH_2Cl_2 2:1); ^1H NMR (500 MHz, $CDCl_3$): δ = 2.52–3.04 (several alkynic singlets), 5.92–6.04 (several aromatic signals), 6.76–8.33, 8.52–8.64, 9.69–9.84 (several complicated aromatic signals); HRMS (MALDI-TOF): *m*/*z* calc. for $C_{86}H_{46}$ 1078.3594 $[M]^+$; found: 1078.3611. The NMR spectra of this compound were very complicated because of the presence of several diastereomeric rotamers in different ratios, so most signals were not readable separately (Figure S8). The solubility of this compound was so low that no signals were observed in the ^{13}C NMR spectrum.

[7]DHA

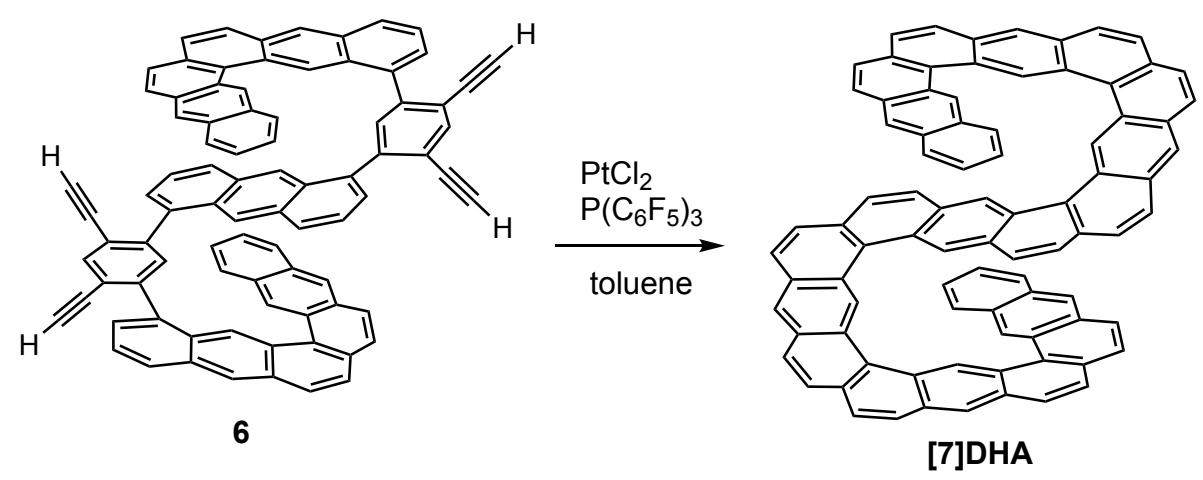

The experimental procedure and the physical and spectroscopic data are provided in Experimental Section.

2. X-ray crystallographic analysis

Diffraction data of compound **[7]DHA** were collected at 93 K on a Pilatus S6M diffractometer with synchrotron radiation (λ = 0.750 Å) equipped in a beamline BL-5A at the High Energy Accelerator Research Organization (KEK), Japan, and processed with XDS. Structures were solved by the intrinsic phasing method (SHELXT 2018) [S4] and refined by non-linear least square method (SHELXL 2018) [S5] using Olex2.1.5 (2023) [S6] as a graphical user interface. Non-hydrogen atoms were refined anisotropically. Hydrogen atoms were refined with the riding model. Additional data are shown in Figures S1–S3.

Crystallographic data of **[7]DHA**: A yellow single crystal was prepared by crystallization from CH_2Cl_2 and hexane. Crystallographic data: Formula $C_{86}H_{46}\cdot 1.01(CH_2Cl_2)\cdot 0.401(C_6H_{14})$, M = 1199.57, monoclinic, $C2_1/c$, a = 23.072(2), b = 12.261(6), c = 21.693(2) Å, β = 105.560(6)°, V = 5912(3) Å^3, Z = 4, D_c = 1.348 g cm^{-3}, θ_{max} = 35.531°, μ(synchrotron) = 0.188 mm^{-1}, number of unique reflections 10915, R_1 = 0.1068 [$I > 2.0\sigma(I)$], wR_2 = 0.4029 (all data), GOF = 1.211, CCDC 2546273.

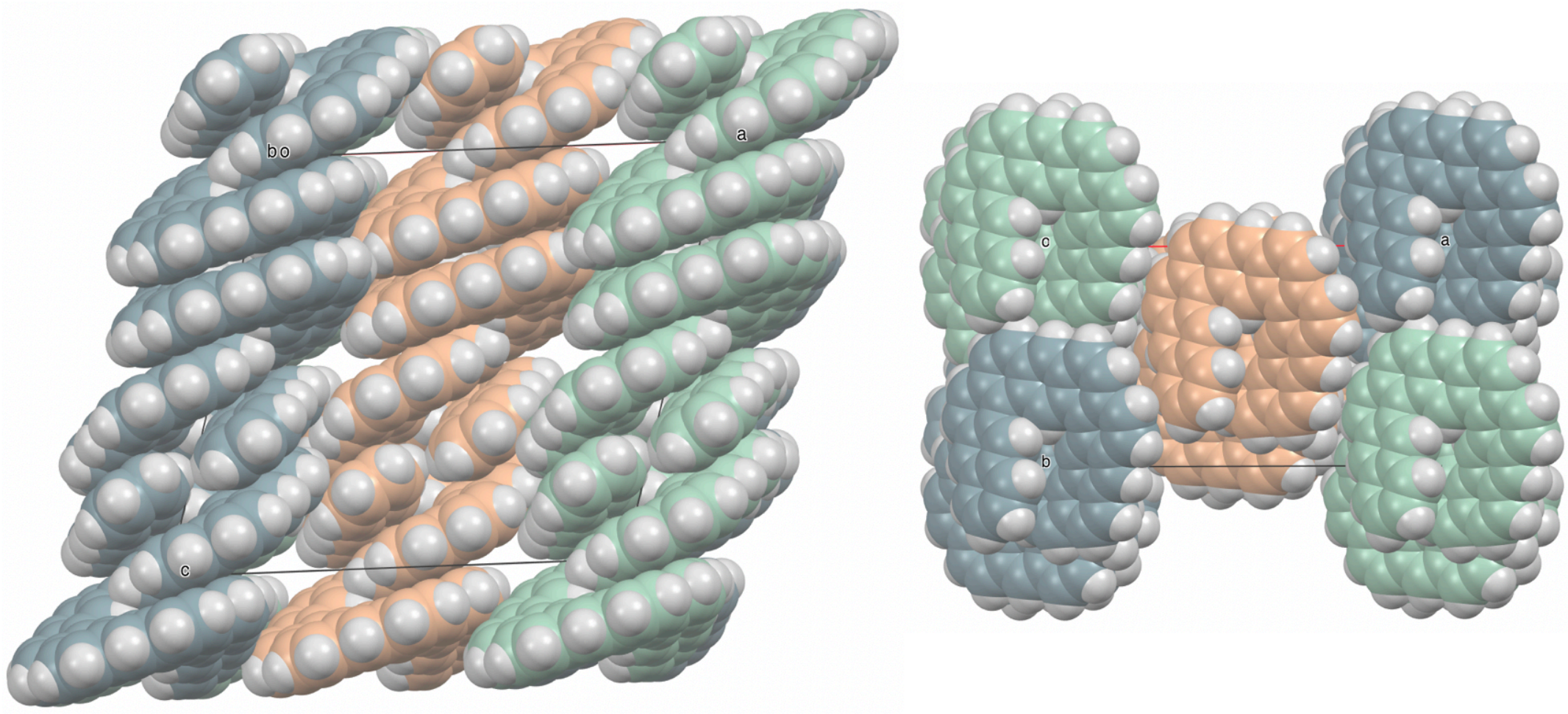

View along the *b* axis　　View along the *c* axis

Figure S1. Packing diagrams of X-ray structure of **[7]DHA** as space-filling models. Solvent molecules are omitted. Molecules along 1D stacking chains are shown in different colors.

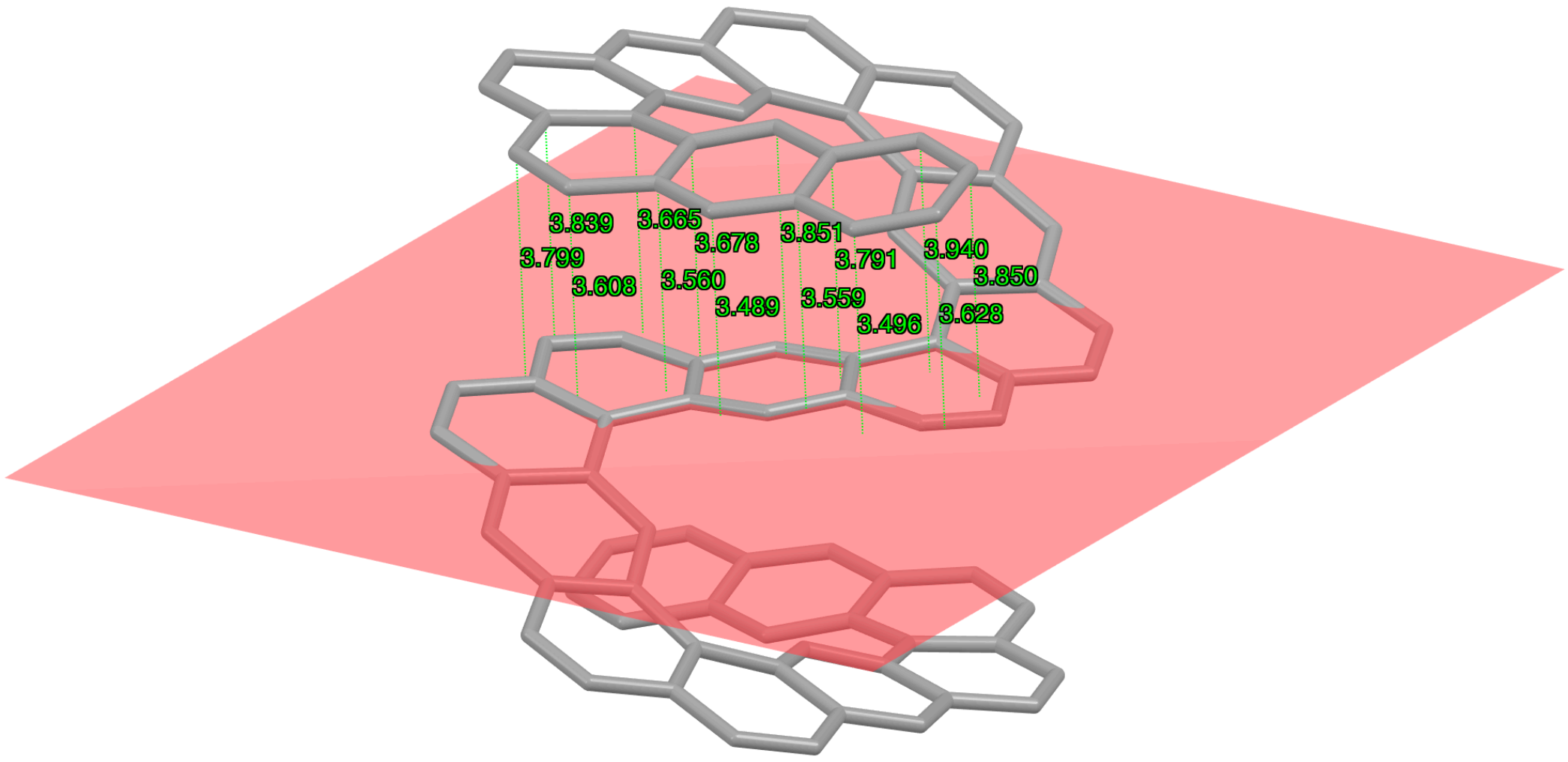

Figure S2. Distances between the averaged plane of the central benzene ring in the central anthracene unit and carbons in the terminal anthracene unit of **[7]DHA** in Å.

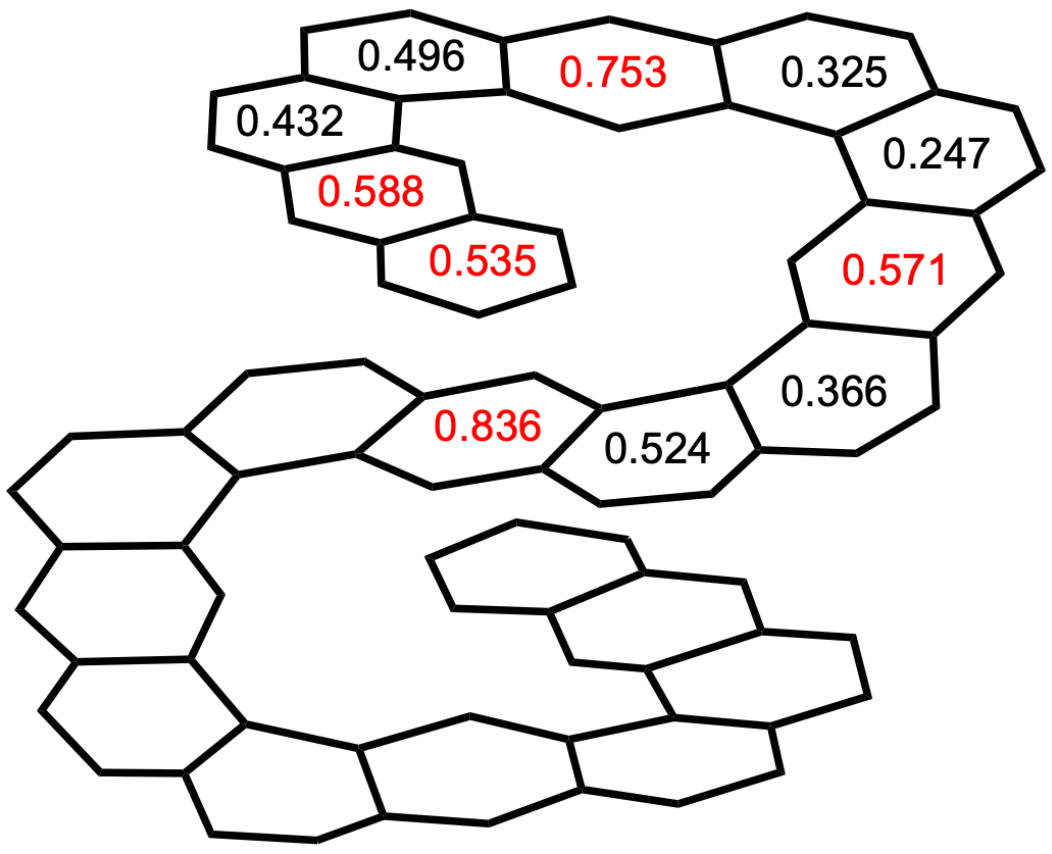

Figure S3. Harmonic oscillator model of aromaticity (HOMA) analysis of C–C bond distances in the X-ray structure of **[7]DHA**. HOMA values are defined by the following equation:
$\mathrm{HOMA} = 1 - (\alpha/n)\sum_{i=1}^{n}(R_{\mathrm{opt}} - R_i)^2$, where α = 257.7 for C–C bond and R_{opt} = 1.388 Å were used, where n is the number of bonds in each ring and R_i the bond length of the i-th bond. Because of the C_i symmetric structure, values are calculated for symmetrically independent benzene rings.

3. Computational methods

Calculations were carried out with Gaussian 16 program [S7] on a windows computer. The structure of **[7]DHA** was optimized by the hybrid DFT method at the B3LYP-D3/6-31G(d,p) level. The frequency analysis was carried out for each optimized structure, giving no imaginary wavenumber for energy minimum structures. The calculations of excited states were carried out by TD-DFT method at the same level with the global minimum structures of **[7]DHA**. The number of excited states (NSTATE) were 40. The calculated UV/vis spectrum and its original data are shown in Figure S4 and Table S1.

NCI analysis: The calculations for NCI analysis were carried out with NCIPLOT 4.0 program. [S8] The input file (wfx. format) for the crystal structure of **[7]DHA** was prepared by the Gaussian program at the B3LYP-D3/6-31G(d,p) level. The 3D RDG isosurface was visualized by using other output files (grad.cube, dens.cube, and vmd formats) with VMD program. [S9]

NICS analysis: The NICS(1) and NICS(−1) values were calculated by GIAO method at GIAO-B3LYP-D3/6-31G(d,p)//B3LYP-D3/6-31G(d,p) level, where the ghost atoms were located at points 1 Å above or below the center of the six-membered rings.[S10] The NICS(1)$_{ave}$ values are averages of the NICS(1) and NICS(−1) values, occasionally adopted for nonplanar aromatic systems.[S11] The NICS(0) were also calculated at the same level, where the ghost atoms were located at the center of the six-membered rings. These results are compiled in Table S2.

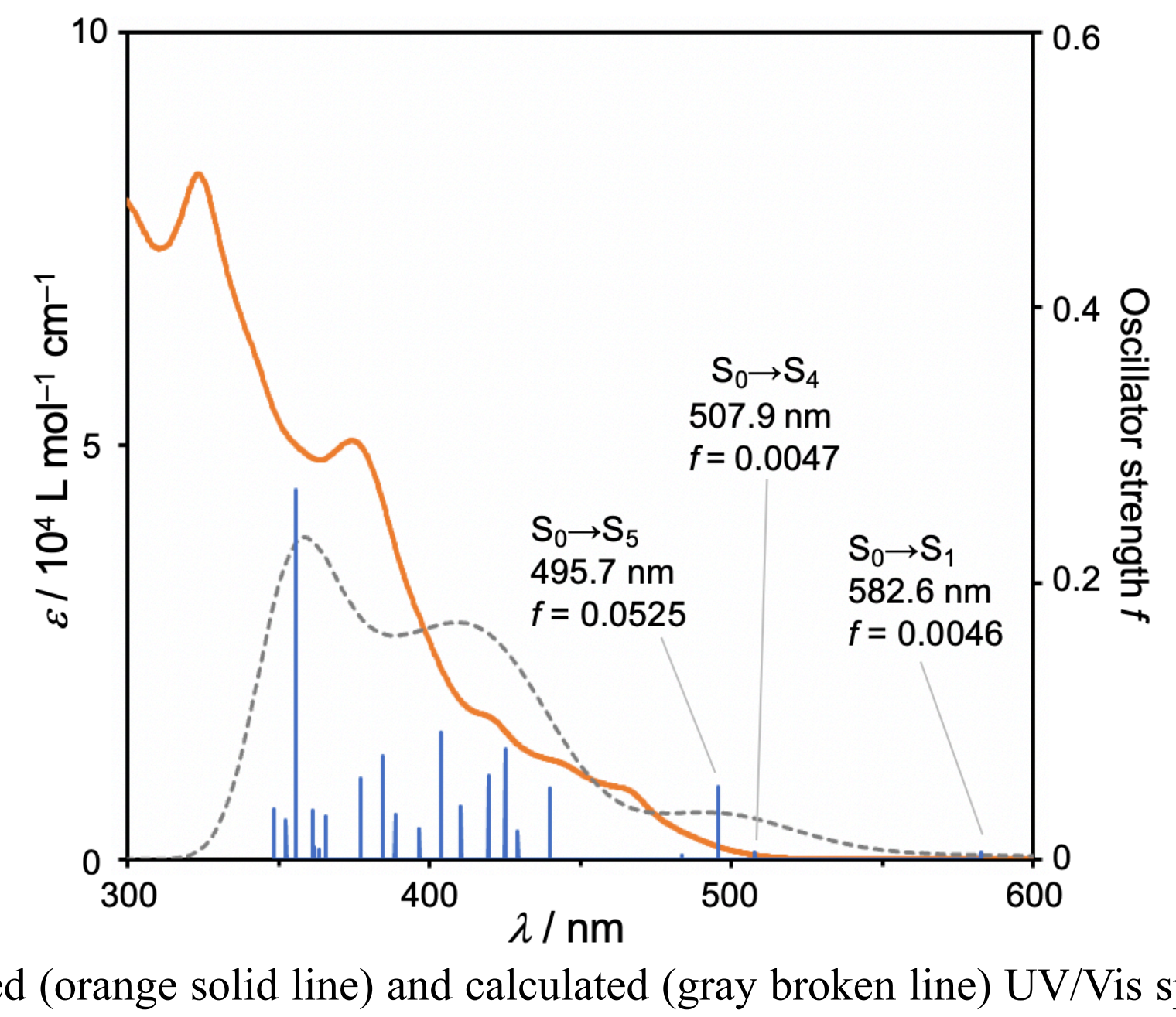

Figure S4. Observed (orange solid line) and calculated (gray broken line) UV/Vis spectra of **[7]DHA**. Observed spectra were measured in $CHCl_3$ at 1.0 × 10^{-5} mol L^{-1}. Calculated spectra were obtained for C_i symmetric structure by TD-DFT calculation at the TD-B3LYP-D3/6-31G(d,p)//B3LYP-D3/6-31G(d,p) level at full width at half maximum 1200 cm^{-1}. Blue bars are oscillator strengths f.

Table S1. Selected TD-DFT data of **[7]DHA** (C_i) calculated at the TD-B3LYP-D3/6-31G(d,p)//B3LYP-D3/6-31G(d,p) level. [a]

excited state	energy/eV	λ/nm [b]	f [c]	transition contribution [d]
1	2.1280	582.63	0.0046	H→L (98.5%)
2	2.2612	548.32	0.0000	H→L+1 (95.6%)
3	2.3376	530.39	0.0000	H–1→L (93.6%)
4	2.4413	507.87	0.0047	H–2→L (44.6%) H→L+2 (51.4%)
5	2.5015	495.65	0.0525	H–2→L (20.7%) H–1→L+1 (47.0%) H→L+2 (29.6%)
6	2.5630	483.75	0.0026	H–2→L (29.9%) H–1→L+1 (50.0%) H→L+2 (17.5%)
7	2.6580	466.45	0.0000	H–2→L+1 (80.8%)
8	2.7249	455.01	0.0000	H–1→L+2 (90.4%)
9	2.7869	444.87	0.0000	H→L+3 (90.9%)
10	2.8179	439.99	0.0509	H–4→L (76.7%)
11	2.8238	439.06	0.0000	H–3→L (79.4%)
12	2.8881	429.29	0.0194	H–2→L+2 (30.0%) H→L+4 (55.5%)
13	2.9163	425.14	0.0798	H–3→L+1 (30.4%) H–2→L+2 (28.9%)
14	2.9551	419.56	0.0605	H–5→L (15.7%) H–3→L+1 (34.5%) H–2→L+2 (25.0%) H→L+4 (15.8%)
15	2.9725	417.11	0.0000	H–4→L+1 (21.7%) H–1→L+4 (10.4%) H→L+5 (44.5%)
16	3.0077	412.22	0.0000	H–4→L+1 (58.8%) H→L+5 (24.0%)
17	3.0209	410.42	0.0383	H–5→L (61.4%) H–3→L+1 (10.7%) H–1→L+3 (12.3%)
18	3.0704	403.81	0.0921	H–3→L+1 (11.1%) H–1→L+3 (69.8%)
19	3.0962	400.44	0.0000	H–6→L (75.4%)
20	3.1257	396.66	0.0222	H→L+6 (70.7%)

a. Forbidden transitions are marked by gray cells.
b. Excitation wavelength.
c. Oscillator strength.
d. H: HOMO, L: LUMO. Only transitions of >10% contributions are listed.

Table S2. NICS values of each benzene ring in **[7]DHA** (C_i) calculated at the GIAO-B3LYP-D3/6-31G(d,p) level. [a]

Ring	NICS(1)	NICS(–1)	NICS(1)$_{av}$ [b]	NICS(0)
A	–12.22	–14.31	–13.26	–11.25
B	–15.34	–16.05	–15.69	–13.80
C	–8.80	–9.19	–9.00	–6.49
D	–8.38	–8.48	–8.43	–6.21
E	–12.70	–12.34	–12.52	–11.21
F	–8.29	–7.75	–8.02	–5.75
G	–7.70	–8.27	–7.98	–5.73
H	–12.46	–12.38	–12.42	–11.14
I	–8.71	–8.05	–8.38	–6.19
J	–10.45	–11.74	–11.09	–8.35
K	–16.95	–16.95	–16.95	–14.45

a. Positions of dummy atoms are shown below. Red: NICS(1), Green: NICS(0), Blue: NICS(–1). Because of the C_i symmetric structure, values of structurally independent benzene rings A–K are calculated.

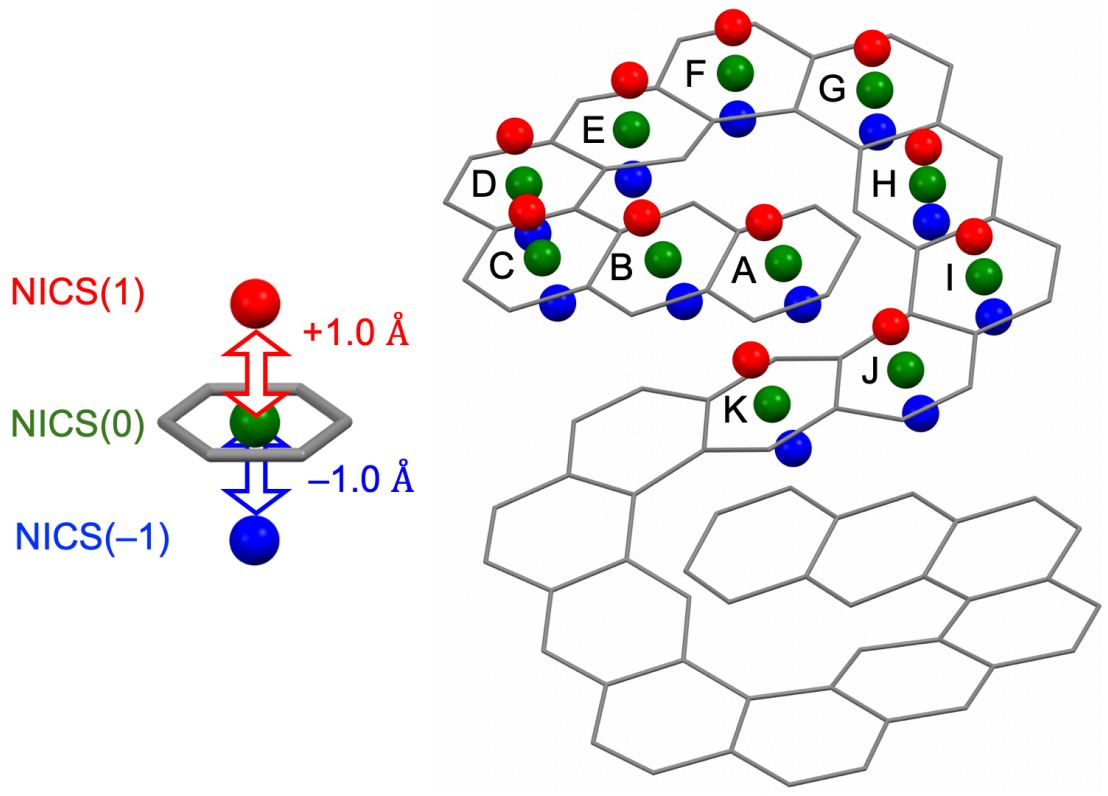

b. Averages of NICS(1) and NICS(–1) values.

Homodesmotic reaction analysis: Thermochemical data of the reactants and the products of the homodesmotic reactions adopted for **[n]DHA** were calculated at the B3LYP-D3/6-31G(d,p) level. [S12,S13] The enthalpies of the homodesmotic reactions (–ΔH_{298}) were calculated from the standard enthalpy of formation of each molecule. The calculated data are listed in Table S3.

Table S3. Thermochemical data of reactants and products of homodesmotic reactions involving **[n]DHA** and enthalpies of reactions calculated at the B3LYP-D3/6-31G(d,p)//B3LYP-D3/6-31G(d,p) level.

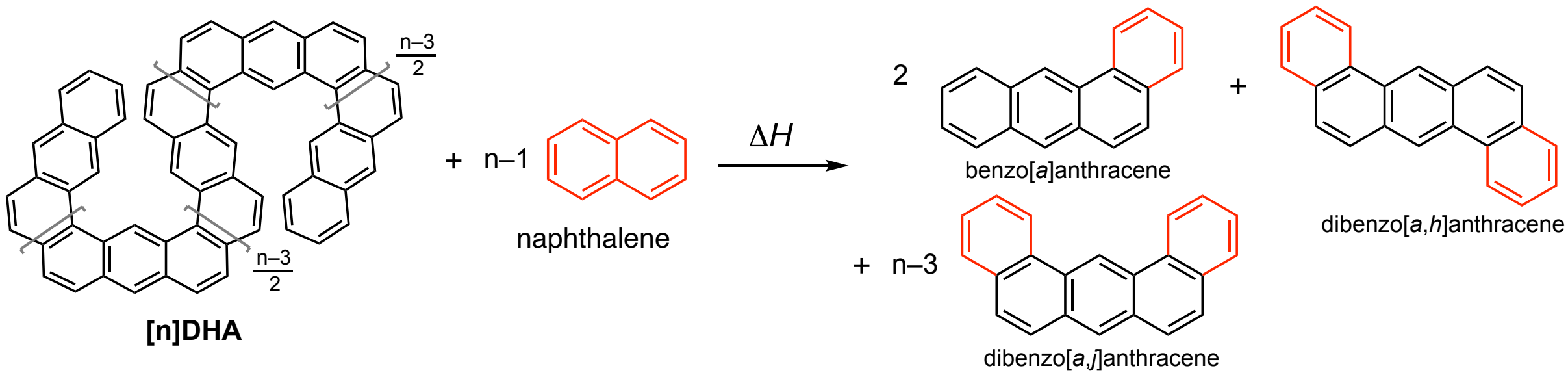

Compound	H_{298} / hartree	Compound	H_{298} / hartree
naphthalene	–385.761208	**[3]DHA** C_i	–1460.964220
benzo[*a*]anthracene	–692.967838	**[3]DHA** C_2	–1460.967528
dibenzo[*a*,*j*]anthracene	–846.575604	**[5]DHA** C_i	–2382.581304
dibenzo[*a*,*h*]anthracene	–846.575838	**[5]DHA** C_2	–2382.591908
		**[7]DHA** C_i	–3304.221088

	$–\Delta H_{298}$/ kJ mol^{-1}
**[3]DHA** C_i	65.32
**[3]DHA** C_2	56.63
**[5]DHA** C_i	96.06
**[5]DHA** C_2	68.22
**[7]DHA** C_i	67.20

1 hartree = 2625.5 kJ mol^{-1}.

Conformational analysis: The X-ray structure was optimized B3LYP-D3/6-31G(d,p) level to give the *P*,*P*,*P*/*M*,*M*,*M* form (EM1). Further calculations were performed by changing the torsion angles along the inner rims using the semiempirical tight-binding scan method at the GFN2-xTB level [S14] with ORCA 6.0.1 program. [S15,S16] Accordingly, two *anti*-forms, *P*,*P*,*M*/*M*,*M*,*M* (EM2) and *P*,*P*,*M*/*M*,*M*,*P* (EM3), were obtained as energy-minimum structures, which were then optimized at the B3LYP-D3/6-31G(d,p) level. For the *syn*-form, an initial was generated from a modelling program. The conformational analysis was performed in a similar manner to give only one energy-minimum structure, *P*,*P*,*M*/*M*,*P*,*P* form (EM4). This structure was then optimized at the B3LYP-D3/6-31G(d,p) level. The calculated structures, structural parameters, and energies are shown in Figure 4 and Tables S4 and S5.

The transition state structures were similarly searched using the the energy-minimum structures by the GFB2-xTB method. However, further optimization of the transition state structures at the B3LYP-D3/6-31G(d,p) level was unsuccessful. Therefore, the following calculations were performed using the GFB2-xTB method. The transition states were searched for the EM1→EM2 and EM2→EM3 processes. Then,

the obtained candidate structures were further optimized to give two structures, TS1 and TS2, the transition states of the first and second inversion processes, respectively. Although final optimization did not fully converge, these structures with one imaginary frequency number were adopted as approximate transition state structures. In contrast, the calculation for the EM3→EM4 process was unsuccessful, presumably because this *anti*→*syn* process involves a considerably unstable transition state. The calculated results are summarized in Figure S5.

Table S4. Torsion angles along [4]helicene inner rims in the calculated energy-minimum structures at the B3LYP-D3/6-31G(d,p) level and the X-ray structures of **[7]DHA**. [a]

**[7]DHA**			Torsion angles /° [d]					
Structure [b]	Conformation [c]	Symmetry	ϕ_{BCD} / ϕ_{CDE}	ϕ_{EFG} / ϕ_{FGH}	ϕ_{HIJ} / ϕ_{IJK}	ϕ_{KLM} / ϕ_{LMN}	ϕ_{NOP} / ϕ_{OPQ}	ϕ_{QRS} / ϕ_{RST}
EM1	*P*,*P*,*P*/*M*,*M*,*M*	C_i	+16.6	+12.8	+13.1	−15.6	−10.8	−11.7
			+11.7	+10.8	+15.6	−13.1	−12.8	−16.6
EM2	*P*,*P*,*M*/*M*,*M*,*M*	C_1	+24.5	+14.6	−2.5	−25.1	−16.5	−12.1
			+8.3	+26.4	−17.8	−12.3	−15.9	−10.6
EM3	*P*,*P*,*M*/*M*,*M*,*P*	C_1	+24.0	+13.7	−5.0	−26.6	−13.7	+4.4
			+9.0	+29.4	−23.2	−9.9	−28.4	+17.5
EM4	*P*,*P*,*M*/*M*,*P*,*P*	C_1	+26.7	+14.1	−1.0	−23.1	+29.3	+12.2
			+11.4	+26.9	−14.1	−5.4	+14.7	+14.7
X-ray	*P*,*P*,*P*/*M*,*M*,*M*	C_i	+18.2	+15.8	+13.9	−18.8	−11.5	−14.3
			+14.3	+11.1	+18.8	−13.9	−15.8	−18.2

a. See Figure 4.
b. EM: Energy-minimum structures.
c. Conformation of each [4]helicene unit: *P* (plus) or *M* (minus).
d. Torsion angles along the inner rim of three benzene units. Each benzene ring is specified as shown right. Cells showing *M* helicity are highlighted in gray.

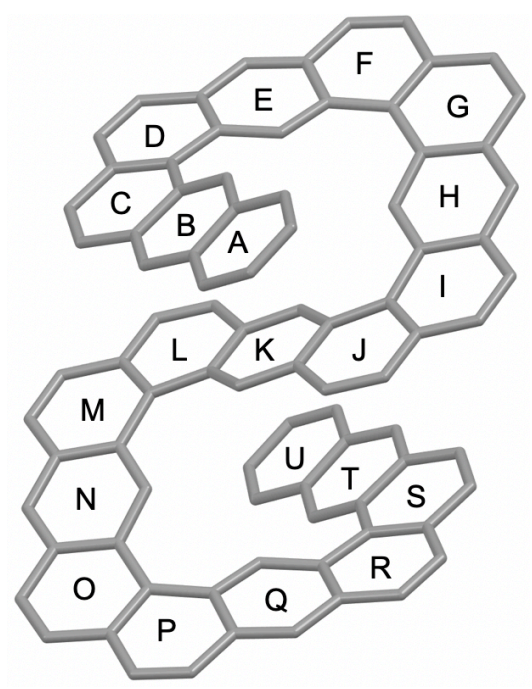

Table S5. Total energies and Gibbs free energies of energy-minimum structures of **[7]DHA** calculated at the B3LYP-D3/6-31G(d,p) level. [a]

	E/ hartree	ΔE /kJ mol^{-1}	G_{298}/ hartree	ΔG_{298}/ kJ mol^{-1}
EM1	−3304.477611	0	−3304.367676	0
EM2	−3304.479183	−6.34	−3304.370171	−6.55
EM3	−3304.481095	−10.39	−3304.369475	−4.72
EM4	−3304.466249	+20.55	−3304.357939	+25.56

a. See Figure 4 and Table S4.

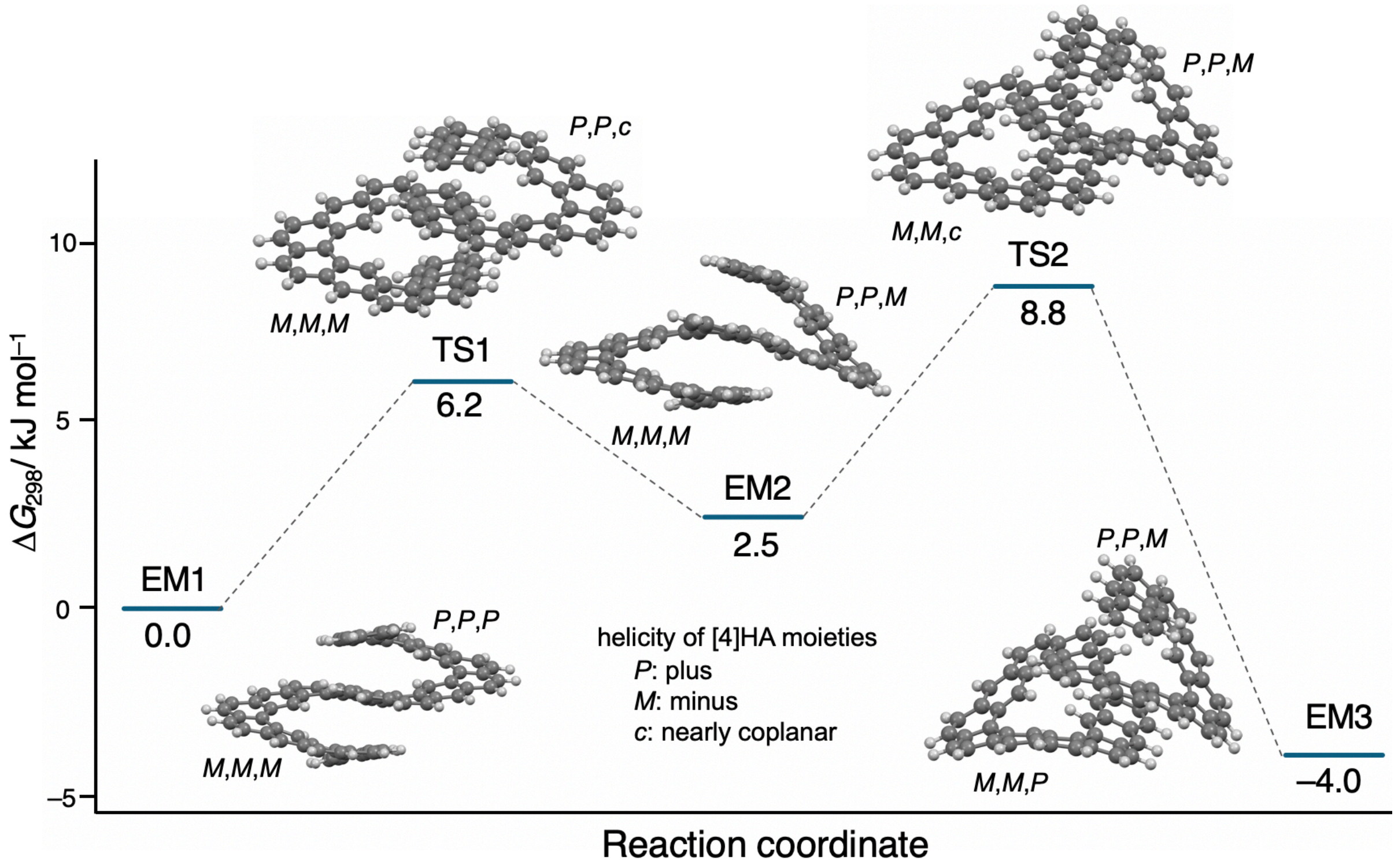

Figure S5. Energy profiles for the helical inversion processes, EM1→EM2 and EM2→EM3, of **[7]DAH** calculated using the GFN2-xTB method. The energy-minimum and transition state structures are shown.

4. References

[S1] K. Xu, Y. Fu, Y. Zhou, F. Hennersdorf, P. Machata, I. Vincon, J. J. Weigand, A. A. Popov, R. Berger, X. Feng, “Cationic Nitrogen-Doped Helical Nanographenes,” *Angewandte Chemie International Edition* **56** (2017): 15876-15881.

[S2] K. Fujise, E. Tsurumaki, G. Fukuhara, N. Hara, Y. Imai, S. Toyota, “Multiple Fused Anthracenes as Helical Polycyclic Aromatic Hydrocarbon Motif for Chiroptical Performance Enhancement,” *Chemistry – An Asian Journal* **15** (2020): 2456-2461.

[S3] K. Fujise, E. Tsurumaki, K. Wakamatsu, S. Toyota, “Construction of Helical Structures with Multiple Fused Anthracenes: Structures and Properties of Long Expanded Helicenes,” *Chemistry – A European Journal* **27** (2021): 4548-4552.

[S4] G. M. Sheldrick, “*SHELXT* – Integrated Space-Group and Crystal-Structure Determination,” *Acta Crystallographica Section A, Foundations and Advances* **A71** (2015): 3-8.

[S5] G. M. Sheldrick, “Crystal Structure Refinement with *SHELXL*,” *Acta Crystallographica Section C, Structural Chemistry* **C71** (2015): 3-8.

[S6] O. V. Dolomanov, L. J. Bourhis, R. J. Gildea, J. A. K. Howard, H. Puschmann, “*OLEX2*: A Complete Structure Solution, Refinement and Analysis Program,” *Journal of Applied Crystallography* **42** (2009): 339-341.

[S7] Gaussian 16, Revision C.01: M. J. Frisch, G. W. Trucks, H. B. Schlegel, G. E. Scuseria, M. A. Robb, J. R. Cheeseman, G. Scalmani, V. Barone, G. A. Petersson, H. Nakatsuji, X. Li, M. Caricato, A. V. Marenich, J. Bloino, B. G. Janesko, R. Gomperts, B. Mennucci, H. P. Hratchian, J. V. Ortiz, A. F. Izmaylov, J. L. Sonnenberg, D. Williams-Young, F. Ding, F. Lipparini, F. Egidi, J. Goings, B. Peng, A. Petrone, T. Henderson, D. Ranasinghe, V. G. Zakrzewski, J. Gao, N. Rega, G. Zheng, W. Liang, M. Hada, M. Ehara, K. Toyota, R. Fukuda, J. Hasegawa, M. Ishida, T. Nakajima, Y. Honda, O. Kitao, H. Nakai, T. Vreven, K. Throssell, J. A. Montgomery, Jr., J. E. Peralta, F. Ogliaro, M. J. Bearpark, J. J. Heyd, E. N. Brothers, K. N. Kudin, V. N. Staroverov, T. A. Keith, R. Kobayashi, J. Normand, K. Raghavachari, A. P. Rendell, J. C. Burant, S. S. Iyengar, J. Tomasi, M. Cossi, J. M. Millam, M. Klene, C. Adamo, R. Cammi, J. W. Ochterski, R. L. Martin, K. Morokuma, O. Farkas, J. B. Foresman, D. J. Fox, Gaussian, Inc., Wallingford CT, 2016.

[S8] R. A. Boto, F. Peccati, R. Laplaza, C. Quan, A. Carbone, J.-P. Piquemal, Y. Maday, J. Contreras-García, “NCIPLOT4: Fast, Robust, and Quantitative Analysis of Noncovalent Interactions,” *Journal of Chemical Theory and Computation* **16** (2020): 4150-4158.

[S9] A. Dalke, K. Schulten, “VMD: Visual Molecular Dynamics,” *Journal of Molecular Graphics* **14** (1996): 33-38.

[S10] Z. Chen, C. S. Wannere, C. Corminboeuf, R. Puchta, P. v. R. Schleyer, “Nucleus-Independent Chemical Shifts (NICS) as an Aromaticity Criterion,” *Chemical Reviews* **105** (2005): 3842–3888.

[S11] J. Cz. Dobrowolski, P. F. J. Lipińskic, “On Splitting of the NICS (1) Magnetic Aromaticity Index,” *RSC Advances* **6** (2016): 23900-23904.

[S12] S. E. Wheeler, “Homodesmotic Reactions for Thermochemistry,” *Wiley Interdisciplinary Reviews: Computational Molecular Science* **2** (2012): 204-220.

[S13] S. E. Wheeler, K. N. Houk, P. v. R. Schleyer, W. D. Allen, “A Hierarchy of Homodesmotic Reactions for Thermochemistry,” *Journal of the American Chemical Society* **131** (2009): 2547-2560.

[S14] C. Bannwarth, S. Ehlert, S. Grimme, “GFN2-xTB—An Accurate and Broadly Parametrized Self-Consistent Tight-Binding Quantum Chemical Method with Multipole Electrostatics and Density-Dependent Dispersion Contributions,” *Journal of Chemical Theory and Computation* **15** (2019): 1652-1671.

[S15] F. Neese, “The ORCA Program System,” *Wiley Interdisciplinary Reviews: Computational Molecular Science* **2** (2012): 73-78.

[S16] F. Neese, “Software Update: The ORCA Program System – Version 6.0,” *Wiley Interdisciplinary Reviews: Computational Molecular Science* **15** (2025): e70019.

5. NMR charts

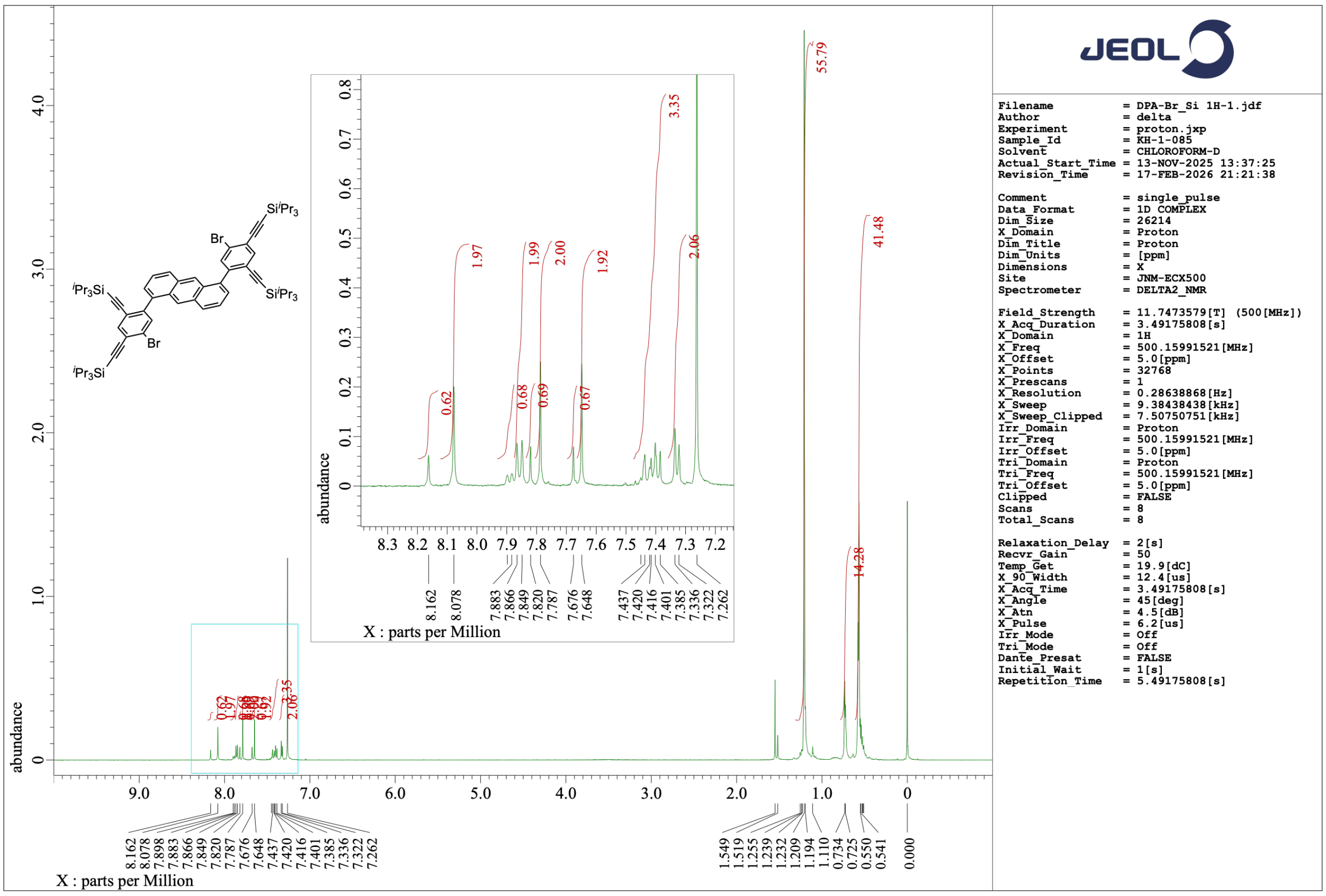

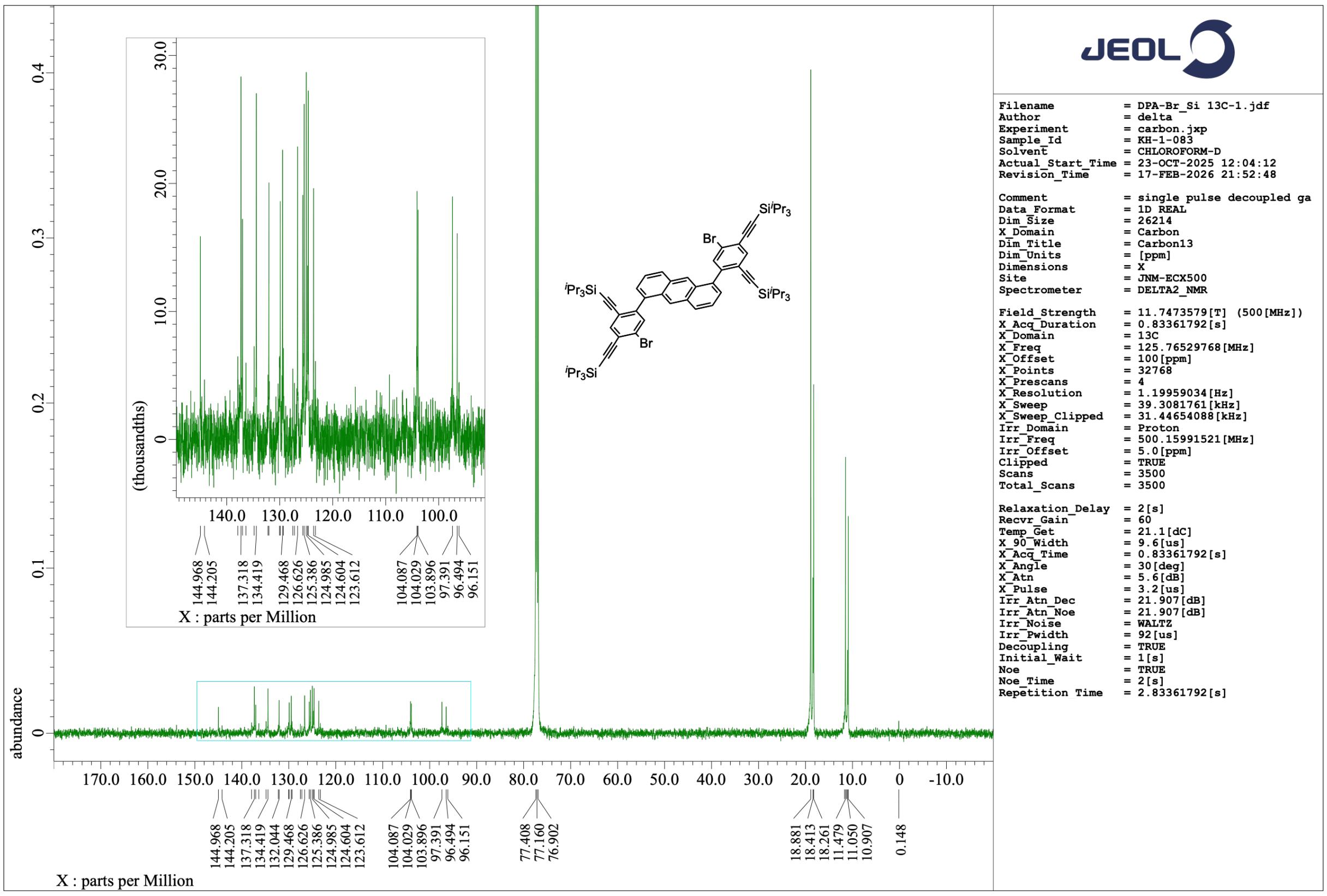

Figure S6. ^{1}H (500 MHz, top) and ^{13}C NMR (125 MHz, bottom) spectra of **3** in $CDCl_3$.

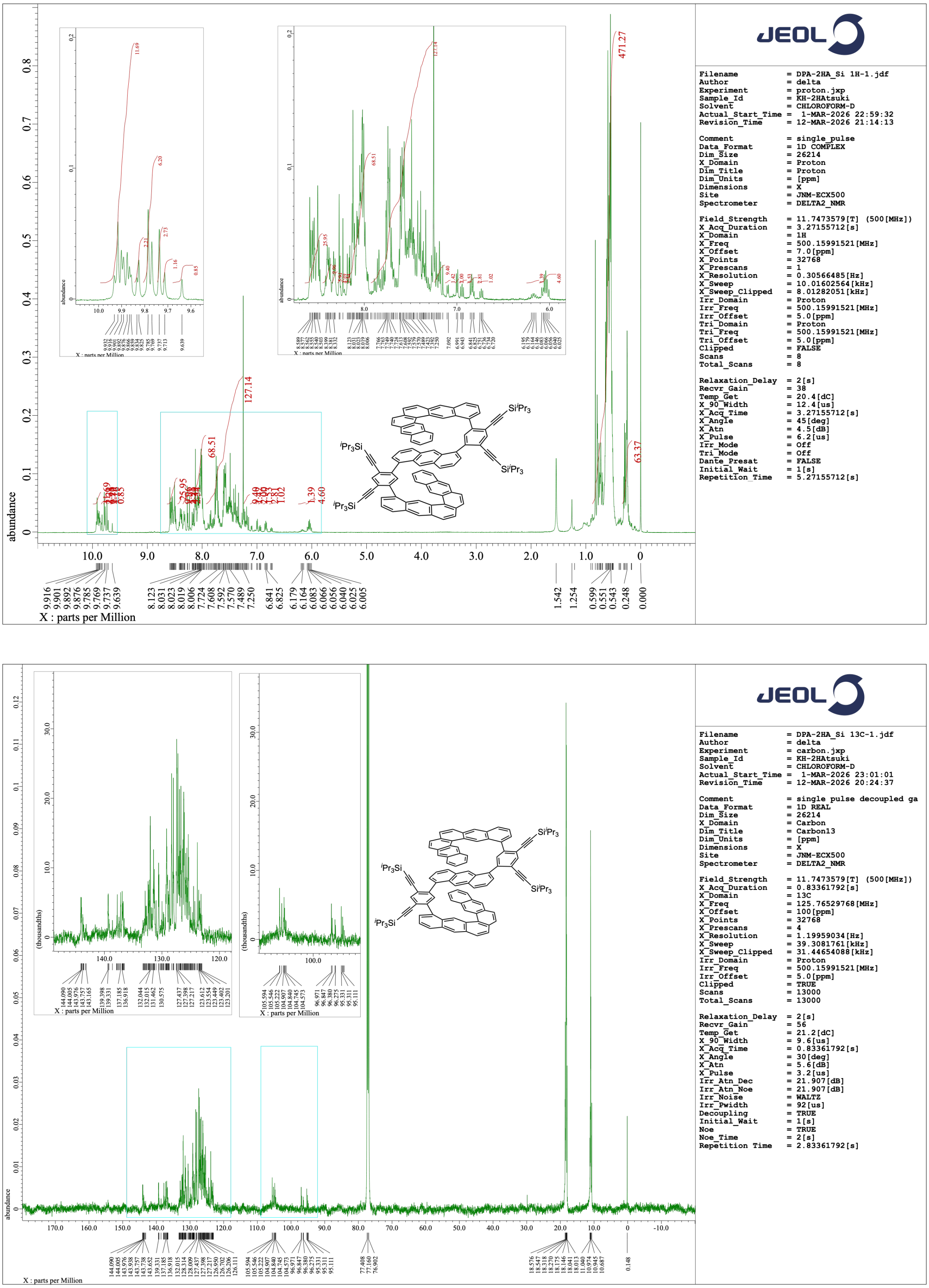

Figure S7. ^{1}H (500 MHz, top) and ^{13}C NMR (125 MHz, bottom) spectra of **5** (a mixture of several rotamers) in $CDCl_3$.

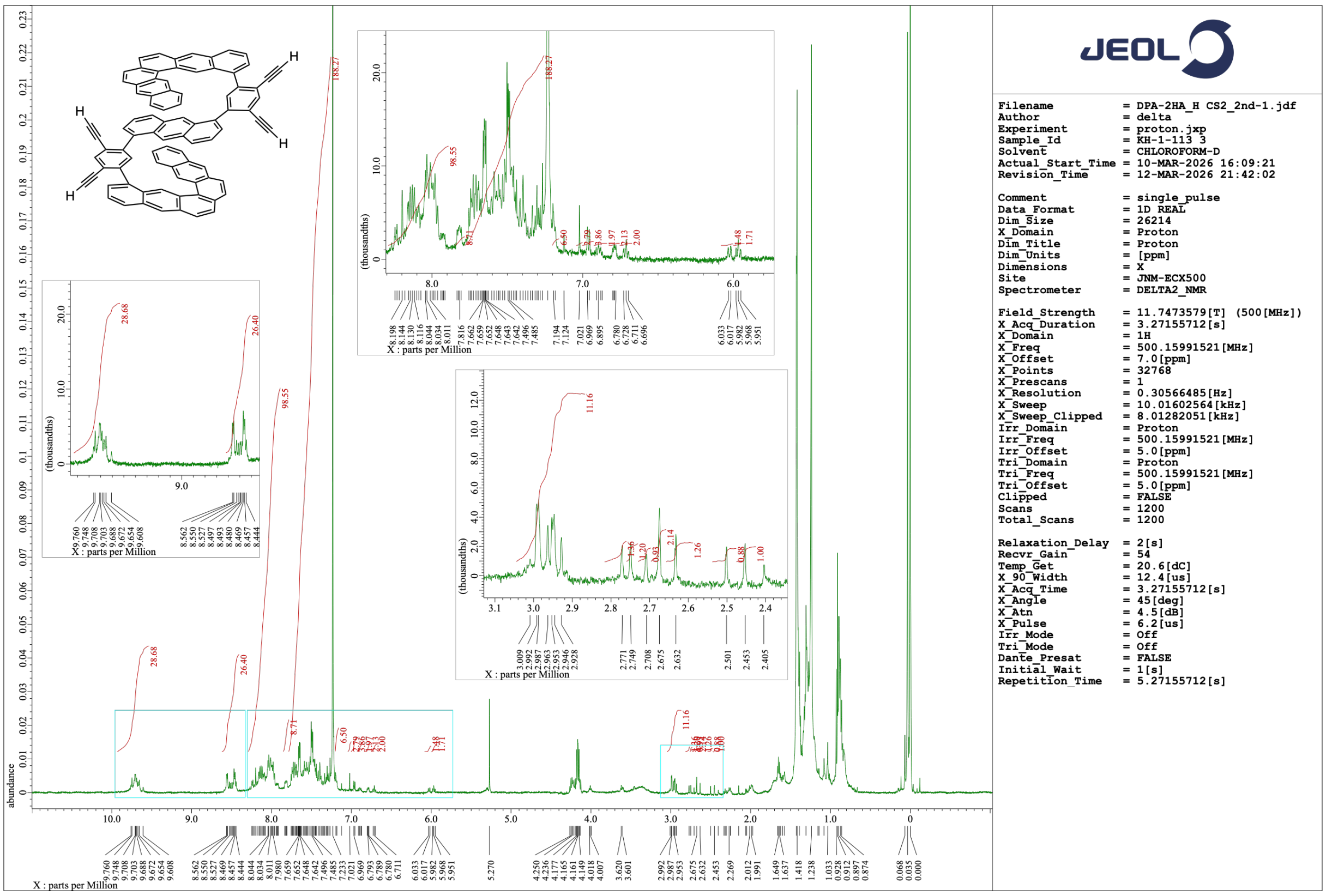

Figure S8. ^{1}H NMR spectrum (500 MHz) of **6** in $CDCl_3/CS_2$ 1:1.

This compound exhibited several pairs of peaks due to the presence of diastereomeric rotamers, where four stereogenic C–C axes exist. The solubility of this compound was so low that there were several signals due to impurities in the compound and in the solvents. The solubility of a pure sample was particularly low. For the same reason, the ^{13}C NMR spectrum could not be measured.

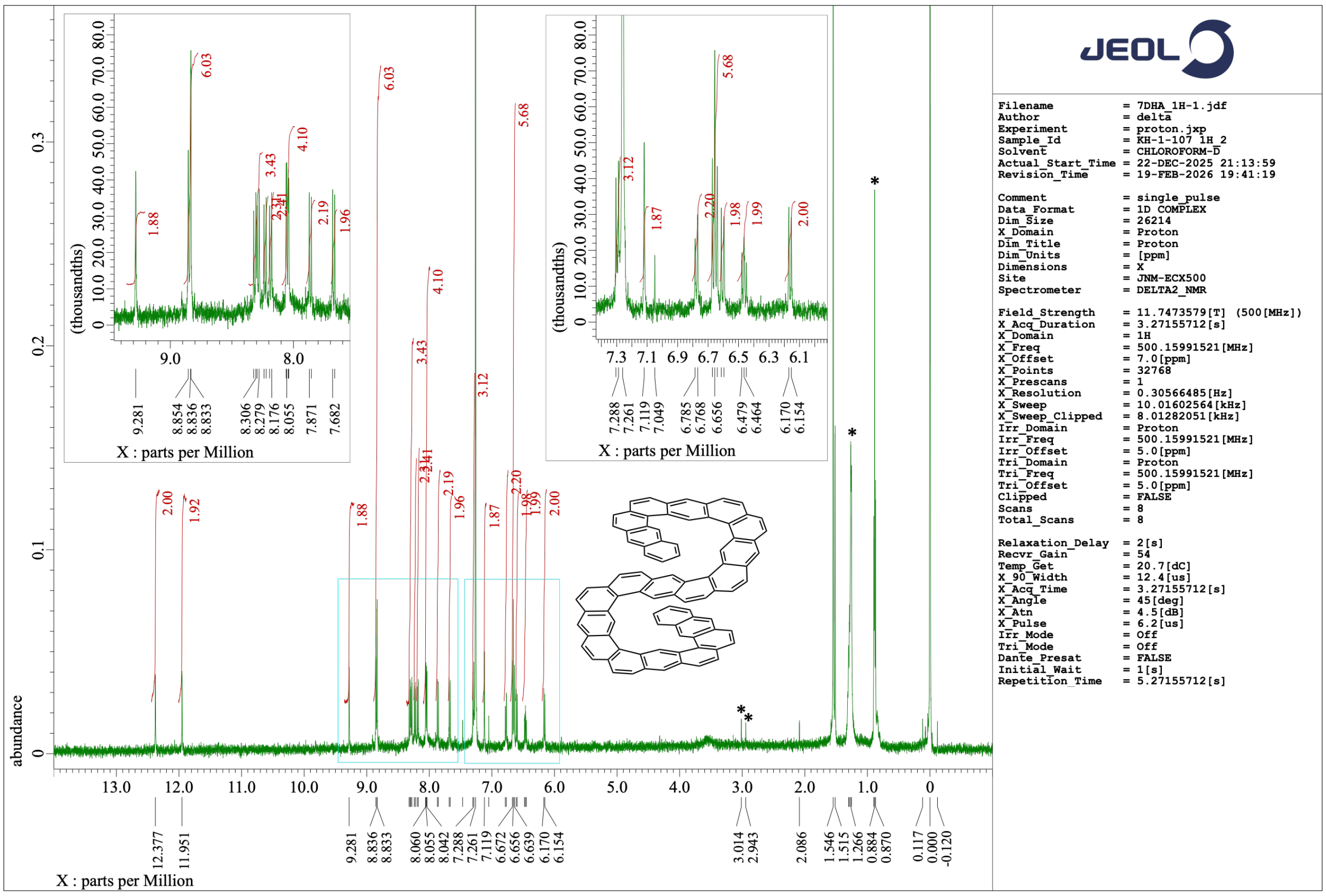

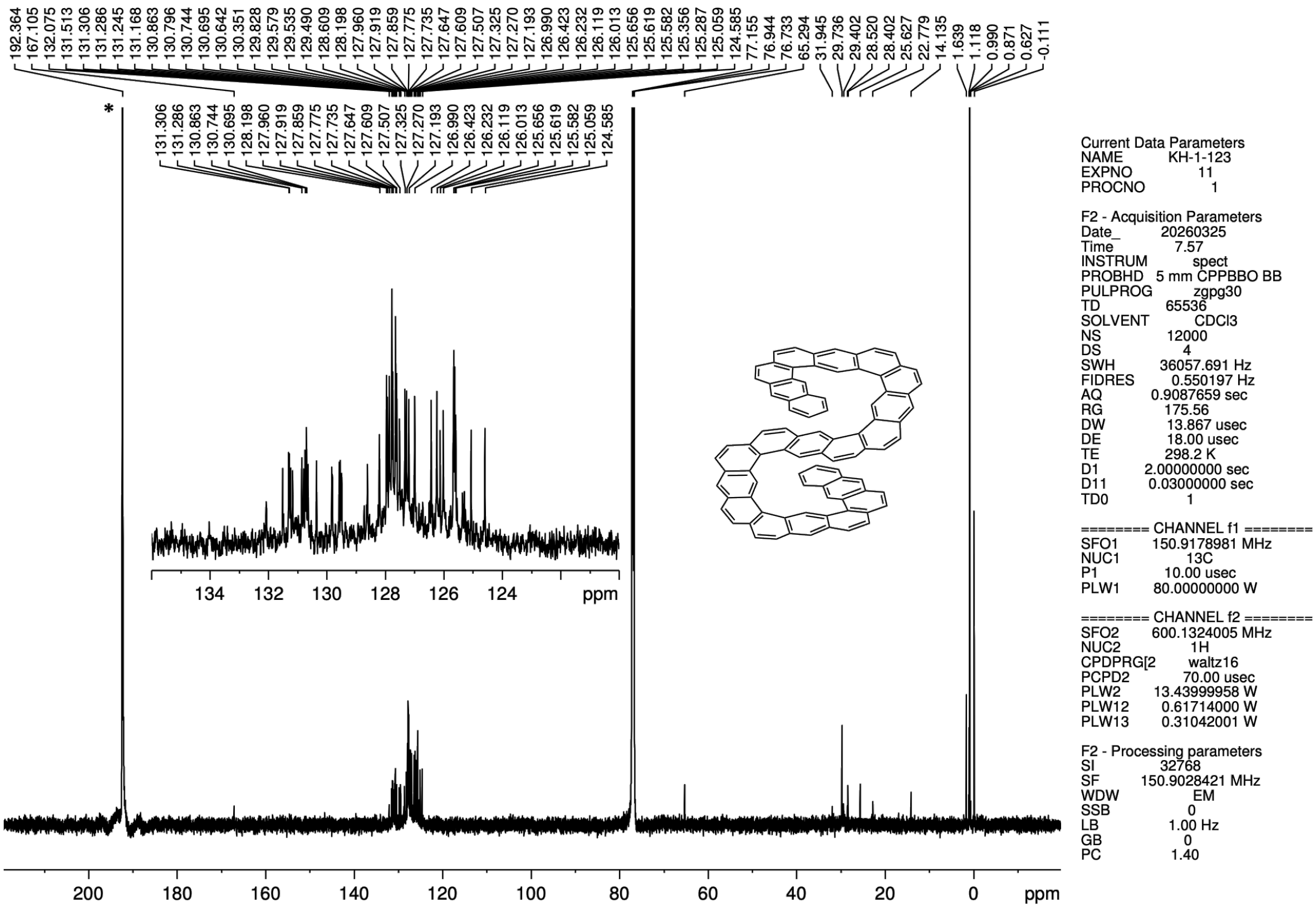

Figure S9. ^{1}H (500 MHz, top) and ^{13}C NMR (151 MHz, bottom) spectra of **[7]DHA** in $CDCl_3$ (*: CS_2 signal).

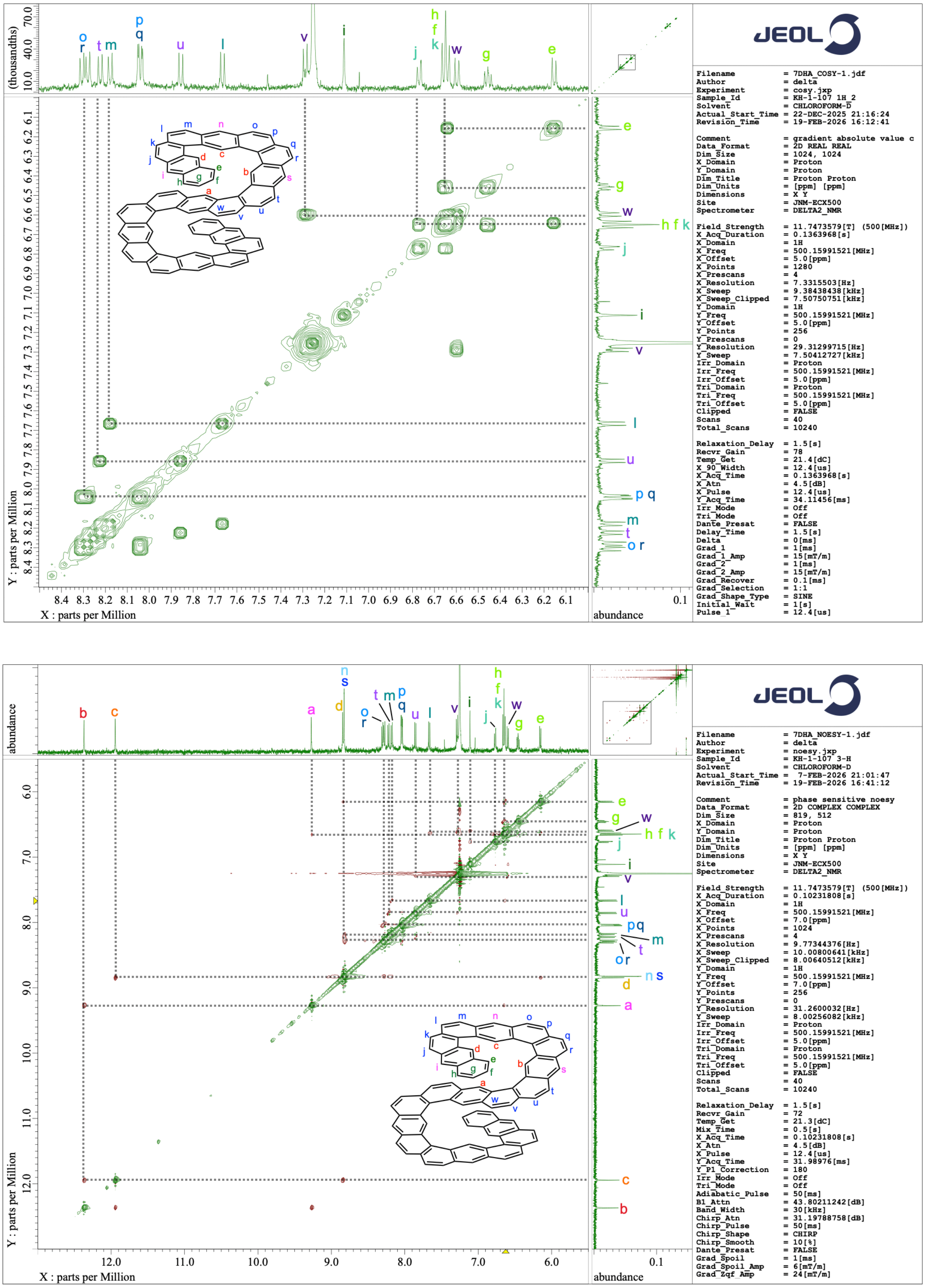

Figure S10. ^{1}H–^{1}H COSY (top) and NOESY (bottom) spectra of **[7]DHA** in $CDCl_3$ at 500 MHz.

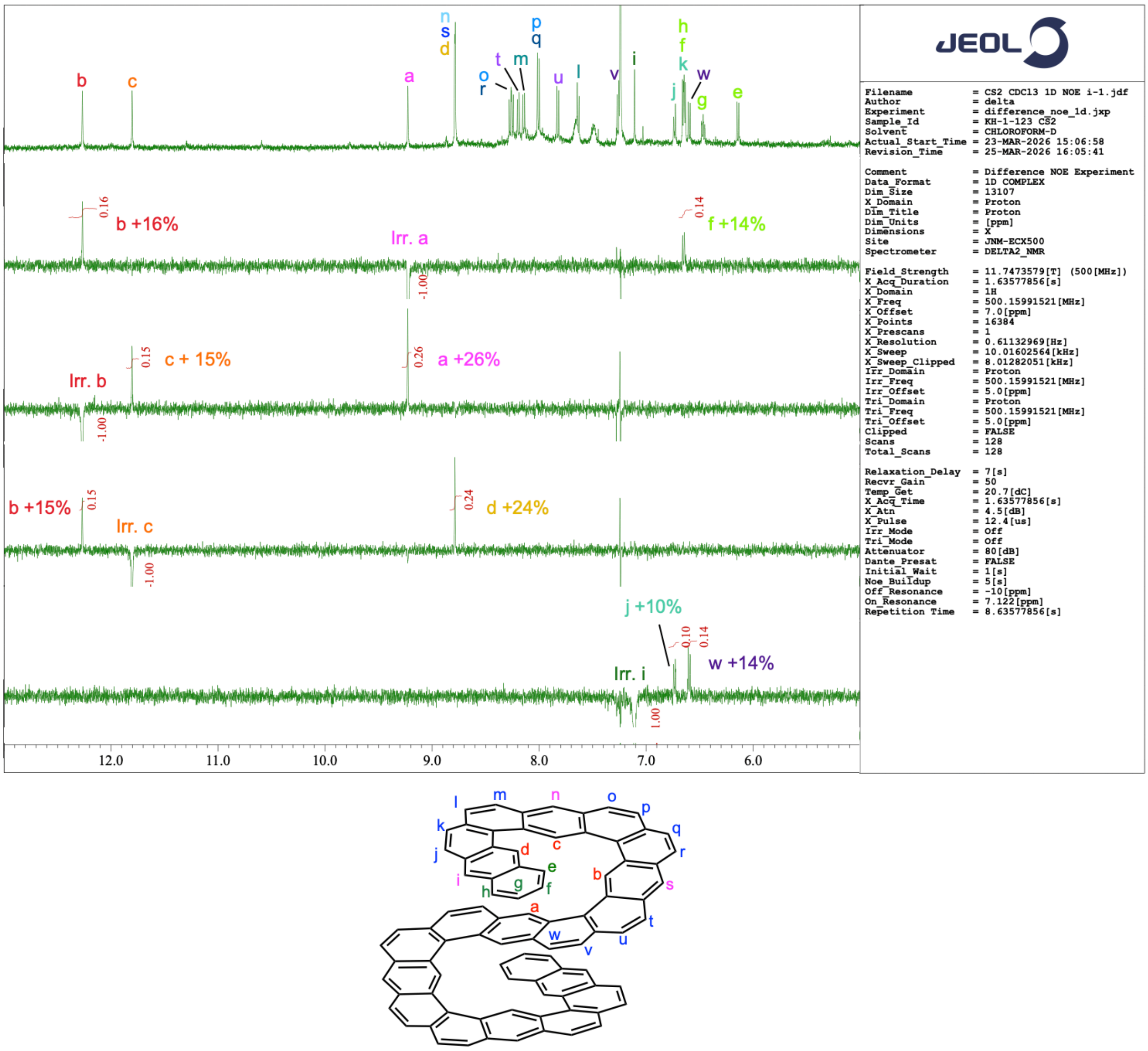

Figure S11. 1D NOE difference spectra of **[7]DHA** at 500 MHz in $CDCl_3$. From top to bottom: no irradiation, irradiation of H_a, irradiation of H_b, irradiation of H_c, and irradiation of H_i.

6. Coordinates of calculated structures

Table S6. Atomic coordinates of calculated structure of (*P*,*P*,*P*/*M*,*M*,*M*)-**[7]DHA** at the B3LYP-D3/6-31G(d,p) level.
Number of imaginary frequency = 0, Total energy = –3304.477611 h

Atom	*x*	*y*	*z*
C	0.6477	–0.9784	0.7593
C	–0.6821	–1.2554	0.2851
C	–1.2584	–0.2548	–0.5051
C	–0.6475	0.9785	–0.7587
C	0.6823	1.2555	–0.2847
C	1.2586	0.2550	0.5056
C	1.3046	2.5323	–0.6225
C	–1.3043	–2.5322	0.6229
C	–0.4679	–3.5334	1.1699
C	–0.9424	–4.8726	1.3573
C	–2.2250	–5.2144	1.0736
C	–3.1564	–4.2089	0.6677
C	–2.7256	–2.8481	0.4734
C	–4.5024	–4.5231	0.4857
C	–5.4711	–3.5657	0.1796
C	–5.1016	–2.1708	0.1004
C	–3.7294	–1.8908	0.2329
C	–6.8128	–3.9815	–0.0716
C	–7.7413	–3.0682	–0.4442
C	–7.4286	–1.6715	–0.5190
C	–6.1461	–1.1711	–0.1750
C	0.4682	3.5335	–1.1695
C	0.9428	4.8727	–1.3570
C	2.2254	5.2144	–1.0733
C	3.1568	4.2088	–0.6674
C	2.7259	2.8481	–0.4731
C	4.5028	4.5229	–0.4855
C	5.4715	3.5654	–0.1796
C	5.1019	2.1706	–0.1005
C	3.7297	1.8907	–0.2329
C	6.8133	3.9811	0.0715
C	7.7417	3.0677	0.4439
C	1.3947	–1.9944	1.4292
C	–1.3945	1.9946	–1.4287
C	–0.8778	3.2421	–1.5604
C	0.8781	–3.2419	1.5609
C	–8.4819	–0.8102	–0.9689
C	–8.3008	0.5276	–1.0746
C	–4.8954	0.9904	0.4028
C	–5.9930	0.2944	–0.1414
C	–7.0718	1.1133	–0.6455
C	–6.9232	2.4990	–0.6946
C	–5.7942	3.1591	–0.2122
C	–4.7566	2.3939	0.4331
C	–5.6634	4.5708	–0.3822
C	–4.5246	5.1952	0.0102
C	–3.4771	4.4826	0.6784
C	–3.6055	3.1088	0.9940
C	–2.2785	5.2082	0.9847
C	–1.2183	4.6026	1.5735
C	–1.3205	3.2495	2.0249
C	–2.5505	2.5090	1.8150
C	–0.2523	2.6717	2.7110
C	–0.3218	1.3800	3.2442
C	–1.5651	0.6710	3.1292
C	–2.6450	1.2688	2.4568
C	0.7771	0.7515	3.9007
C	0.6550	–0.5187	4.4029
C	–0.5736	–1.2273	4.2807
C	–1.6560	–0.6453	3.6716
C	8.3009	–0.5283	1.0740
C	7.0718	–1.1138	0.6451
C	5.9930	–0.2948	0.1412
C	6.1463	1.1708	0.1748
C	7.4289	1.6710	0.5187
C	8.4821	0.8096	0.9685
C	6.9231	–2.4995	0.6941
C	5.7939	–3.1594	0.2118
C	4.7564	–2.3941	–0.4333
C	4.8953	–0.9906	–0.4030
C	5.6628	–4.5711	0.3818
C	4.5239	–5.1953	–0.0104
C	3.4765	–4.4825	–0.6786
C	3.6051	–3.1087	–0.9941
C	2.2778	–5.2080	–0.9848
C	1.2176	–4.6022	–1.5735
C	1.3200	–3.2491	–2.0249
C	2.5501	–2.5088	–1.8152
C	0.2519	–2.6712	–2.7110
C	0.3215	–1.3795	–3.2442
C	1.5650	–0.6707	–3.1293
C	2.6448	–1.2686	–2.4570
C	–0.7773	–0.7508	–3.9006
C	–0.6551	0.5194	–4.4028
C	0.5737	1.2279	–4.2806
C	1.6561	0.6456	–3.6717
H	–2.2038	–0.4179	–0.9978
H	2.2041	0.4181	0.9983
H	–0.2378	–5.6161	1.7194
H	–2.5757	–6.2357	1.1934
H	–4.8141	–5.5599	0.5898
H	–3.4137	–0.8761	0.1423
H	–7.0592	–5.0366	0.0069
H	–8.7552	–3.3763	–0.6833
H	0.2382	5.6162	–1.7191
H	2.5761	6.2357	–1.1931

H	4.8146	5.5597	–0.5896
H	3.4138	0.8760	–0.1423
H	7.0598	5.0362	–0.0069
H	8.7557	3.3756	0.6831
H	2.4043	–1.7694	1.7582
H	–2.4041	1.7696	–1.7575
H	–1.4745	4.0434	–1.9850
H	1.4747	–4.0433	1.9856
H	–9.4284	–1.2647	–1.2475
H	–9.0884	1.1761	–1.4479
H	–4.1044	0.4200	0.8376
H	–7.7256	3.0910	–1.1290
H	–6.4692	5.1176	–0.8638
H	–4.3893	6.2594	–0.1628
H	–2.2218	6.2508	0.6838
H	–0.2857	5.1356	1.7326
H	0.6628	3.2465	2.8302
H	–3.5831	0.7341	2.4800
H	1.7153	1.2941	3.9815
H	1.5007	–0.9980	4.8868
H	–0.6446	–2.2418	4.6601
H	–2.5877	–1.1929	3.5592
H	9.0884	–1.1769	1.4472
H	9.4287	1.2639	1.2471
H	7.7254	–3.0916	1.1284
H	4.1043	–0.4201	–0.8376
H	6.4686	–5.1180	0.8633
H	4.3885	–6.2595	0.1626
H	2.2209	–6.2505	–0.6839
H	0.2849	–5.1350	–1.7325
H	–0.6634	–3.2457	–2.8301
H	3.5831	–0.7341	–2.4803
H	–1.7156	–1.2931	–3.9814
H	–1.5007	0.9989	–4.8866
H	0.6448	2.2424	–4.6600
H	2.5878	1.1931	–3.5593
