# Supplementary material for: Synthesis and Structure of an S‐Shaped Double Expanded Helicene With Seven Fused Anthracene Units
Source: Chemistry. 2026 Jun 8;32(35):e71249. doi: 10.1002/chem.71249 (PMC13587644; doi:10.1002/chem.71249)

checkCIF/PLATON report

Structure factors have been supplied for datablock(s) kh-1-107-2-4

THIS REPORT IS FOR GUIDANCE ONLY. IF USED AS PART OF A REVIEW PROCEDURE FOR PUBLICATION, IT SHOULD NOT REPLACE THE EXPERTISE OF AN EXPERIENCED CRYSTALLOGRAPHIC REFEREE.

No syntax errors found. CIF dictionary Interpreting this report

Datablock: kh-1-107-2-4

Bond precision:	C-C = 0.0059 A		Wavelength=3/4
Cell:	a=23.072(2)	b=12.261(6)	c=21.693(2)
	alpha=90	beta=105.560(6)	gamma=90
Temperature:	95 K		

	Calculated	Reported
Volume	5912(3)	5912(3)
Space group	C 2/c	C 1 2/c 1
Hall group	-C 2yc	-C 2yc
Moiety formula	C86 H46, 0.401(C6 H14), 1.01(C H2 Cl2)	C86 H46, 1.01(C H2 Cl2), 0.401(C6 H14)
Sum formula	C89.42 H53.63 Cl2.02	C89.42 H53.63 Cl2.02
Mr	1199.56	1199.57
Dx,g cm-3	1.348	1.348
Z	4	4
Mu (mm-1)	0.186	0.188
F000	2497.9	2498.0
F000'	2500.20	
h,k,lmax	35,18,33	35,18,33
Nref	11512	10915
Tmin,Tmax	0.985,0.996	
Tmin'	0.985	

Correction method= Not given

```
Data completeness= 0.948          Theta(max)= 35.531

                                                    wR2(reflections)=
R(reflections)= 0.1068( 4859)                       0.4029( 10915)

S = 1.211                  Npar= 445
```

The following ALERTS were generated. Each ALERT has the format
test-name_ALERT_alert-type_alert-level.
Click on the hyperlinks for more details of the test.

Alert level B

```
PLAT084_ALERT_3_B High wR2 Value (i.e. > 0.25) ...................       0.40 Report
PLAT230_ALERT_2_B Hirshfeld Test Diff for    C4        --C00E     .        8.3 s.u.
PLAT230_ALERT_2_B Hirshfeld Test Diff for    C00S      --C016     .        7.7 s.u.
PLAT230_ALERT_2_B Hirshfeld Test Diff for    C00X      --C013     .        7.2 s.u.
PLAT410_ALERT_2_B Short Intra H...H Contact  H003      ..H3       .       1.83 Ang.
                                                1-x,1-y,1-z  =      5_666 Check
PLAT410_ALERT_2_B Short Intra H...H Contact  H006      ..H00F     .       1.83 Ang.
                                                      x,y,z  =      1_555 Check
PLAT410_ALERT_2_B Short Intra H...H Contact  H3        ..H00F     .       1.81 Ang.
                                                      x,y,z  =      1_555 Check
```

Alert level C

```
PLAT026_ALERT_3_C Ratio Observed / Unique Reflections (too) Low ..        45% Check
PLAT042_ALERT_1_C Calc. and Reported MoietyFormula Strings  Differ     Please Check
              Calc: C86 H46, 0.401(C6 H14), 1.01(C H2 Cl2)
              Rep.: C86 H46, 1.01(C H2 Cl2), 0.401(C6 H14)
PLAT052_ALERT_1_C Info on Absorption Correction Method   Not Given     Please Do !
PLAT082_ALERT_2_C High R1 Value ..................................       0.11 Report
PLAT230_ALERT_2_C Hirshfeld Test Diff for    C5        --C00I     .        7.0 s.u.
PLAT241_ALERT_2_C High   'MainMol' Ueq as Compared to Neighbors of       C018 Check
PLAT260_ALERT_2_C Large Average Ueq of Residue Including        C7      0.125 Check
PLAT340_ALERT_3_C Low Bond Precision on  C-C Bonds ...............    0.00587 Ang.
PLAT411_ALERT_2_C Short Inter H...H Contact  H012      ..H012     .       2.13 Ang.
                                              1-x,y,1/2-z  =      2_655 Check
PLAT906_ALERT_3_C Large K Value in the Analysis of Variance ......     25.397 Check
PLAT906_ALERT_3_C Large K Value in the Analysis of Variance ......      5.433 Check
PLAT906_ALERT_3_C Large K Value in the Analysis of Variance ......      2.594 Check
PLAT911_ALERT_3_C Missing FCF Refl Between Thmin & STh/L=    0.600         34 Report
              -24  0  2,   2  0  2,   4  2  2,  16  0  2,  18  0  2,  -6  0  4,
               -2  2  4,   3  1  4,  14  0  4, -15  7  5,  -9 11  5,  -2  6  5,
                1  1  5,   6  2  5,  -4  0  6,  10  0  6,  12  0  6,  16  0  6,
               16  2  6, -10  2  7,  -8  4  8,  -5 11  9, -24  0 10,   0  0 10,
              -24  0 12, -10  0 12,   0  0 12,  -9  1 13, -16  2 18,  -8  0 18,
                   (    4 More Missing: see the .ckf listing file)
PLAT934_ALERT_3_C Number of (Iobs-Icalc)/Sigma(W) > 10 Outliers ..          1 Check
               -2  6  3,
```

Alert level G

```
ABSMU01_ALERT_1_G  Calculation of _exptl_absorpt_correction_mu
                not performed for this radiation type.
```

```
PLAT068_ALERT_1_G Reported F000 Differs from Calcd (or Missing)...     Please Check
PLAT072_ALERT_2_G SHELXL First  Parameter in WGHT  Unusually Large       0.20 Report
PLAT302_ALERT_4_G Anion/Solvent/Minor-Residue Disorder (Resd    2)       100% Note
PLAT302_ALERT_4_G Anion/Solvent/Minor-Residue Disorder (Resd    3)       100% Note
PLAT304_ALERT_4_G Non-Integer Number of Atoms in ..... (Resd    2)       8.02 Check
PLAT304_ALERT_4_G Non-Integer Number of Atoms in ..... (Resd    3)       2.53 Check
PLAT333_ALERT_2_G Large Aver C6-Ring C-C Dist C4       -C00J     .       1.43 Ang.
PLAT413_ALERT_2_G Short Inter XH3 .. XHn     H00S     ..H16C     .       2.11 Ang.
                                     1/2-x,3/2-y,1-z  =      7_566 Check
PLAT720_ALERT_4_G Number of Unusual/Non-Standard Labels ..........         62 Note
              C003    H003    C005    C006    H006    C009    C00A    C00C
              H00C    C00E    C00F    H00F    C00G    C00H    H00H    C00I
              C00J    C00K    C00L    C00M    C00N    C00O    C00P    H00P
              C00Q    C00R    H00R    C00S    H00S    C00T    C00U    C00V
              H00V    C00W    H00W    C00X    C00Y    H00Y    C00Z    H00Z
              C010    H010    C011    H011    C012    H012    C013    H013
              C014    H014    C015    H015    C016    H016    C017    H017
              C018    H018    C019    H019    H6AA    H6AB
PLAT764_ALERT_4_G Overcomplete CIF Bond List Detected (Rep/Expd) .       1.14 Ratio
PLAT779_ALERT_4_G Suspect or Irrelevant (Bond) Angle(s) in CIF ...      39.80 Deg.
              C6A  -CL2  -C8      1_555   1_555   1_555 ......     #  130 Check
PLAT779_ALERT_4_G Suspect or Irrelevant (Bond) Angle(s) in CIF ...      27.90 Deg.
              C7   -CL2  -C6A     1_555   1_555   1_555 ......     #  131 Check
PLAT779_ALERT_4_G Suspect or Irrelevant (Bond) Angle(s) in CIF ...      42.00 Deg.
              C7   -CL2  -C8      1_555   1_555   1_555 ......     #  132 Check
PLAT779_ALERT_4_G Suspect or Irrelevant (Bond) Angle(s) in CIF ...      44.90 Deg.
              C6A  -CL1A -C8      1_555   1_555   1_555 ......     #  133 Check
PLAT779_ALERT_4_G Suspect or Irrelevant (Bond) Angle(s) in CIF ...      30.60 Deg.
              C6A  -CL1A -C7      1_555   1_555   1_555 ......     #  134 Check
PLAT779_ALERT_4_G Suspect or Irrelevant (Bond) Angle(s) in CIF ...      32.40 Deg.
              C7   -C6A  -CL2     1_555   1_555   1_555 ......     #  150 Check
PLAT779_ALERT_4_G Suspect or Irrelevant (Bond) Angle(s) in CIF ...      37.10 Deg.
              C6A  -C8   -C7      1_555   1_555   1_555 ......     #  170 Check
PLAT779_ALERT_4_G Suspect or Irrelevant (Bond) Angle(s) in CIF ...      28.00 Deg.
              C7   -C8   -CL2     1_555   1_555   1_555 ......     #  177 Check
PLAT779_ALERT_4_G Suspect or Irrelevant (Bond) Angle(s) in CIF ...      39.10 Deg.
              CL2  -C7   -H7B     1_555   1_555   1_555 ......     #  184 Check
PLAT779_ALERT_4_G Suspect or Irrelevant (Bond) Angle(s) in CIF ...      36.60 Deg.
              CL1A -C16  -H16B    1_555   1_555   1_555 ......     #  205 Check
PLAT779_ALERT_4_G Suspect or Irrelevant (Bond) Angle(s) in CIF ...      35.30 Deg.
              C6A  -C16  -C7      1_555   1_555   1_555 ......     #  207 Check
PLAT789_ALERT_4_G Atoms with Negative _atom_site_disorder_group  #          5 Check
PLAT822_ALERT_4_G CIF-embedded .res Contains Negative PART Numbers          1 Check
PLAT912_ALERT_4_G Missing # of FCF Reflections Above STh/L=  0.600        563 Note
PLAT930_ALERT_2_G FCF-based   Twin Law         [ 1 0 4] Est.d BASF       0.06 Check
PLAT931_ALERT_5_G CIFcalcFCF  Twin Law         [ 1 0 4] Est.d BASF       0.06 Check
PLAT933_ALERT_2_G Number of HKL-OMIT Records in Embedded .res File          7 Note
              -10  2  7,  -2  2  4,  -2  6  5,   4  2  2,   6  2  5,  16  0  2,
               16  2  6,
PLAT941_ALERT_3_G Average HKL Measurement Multiplicity ..........         2.9 Low
PLAT969_ALERT_5_G The 'Henn et al.' R-Factor-gap value ..........       8.273 Note
              Predicted wR2: Based on SigI**2  4.87 or SHELX Weight 33.28
PLAT978_ALERT_2_G Number C-C Bonds with Positive Residual Density.          5 Info
```

---

0 **ALERT level A** = Most likely a serious problem - resolve or explain

7 **ALERT level B** = A potentially serious problem, consider carefully

```
14 ALERT level C = Check. Ensure it is not caused by an omission or oversight
31 ALERT level G = General information/check it is not something unexpected

 4 ALERT type 1 CIF construction/syntax error, inconsistent or missing data
17 ALERT type 2 Indicator that the structure model may be wrong or deficient
 9 ALERT type 3 Indicator that the structure quality may be low
20 ALERT type 4 Improvement, methodology, query or suggestion
 2 ALERT type 5 Informative message, check
```

It is advisable to attempt to resolve as many as possible of the alerts in all categories. Often the minor alerts point to easily fixed oversights, errors and omissions in your CIF or refinement strategy, so attention to these fine details can be worthwhile. It is up to the individual to critically assess their own results and, if necessary, seek expert advice.

PLATON version of 26/09/2025; check.def file version of 20/09/2025

Datablock kh-1-107-2-4 - ellipsoid plot

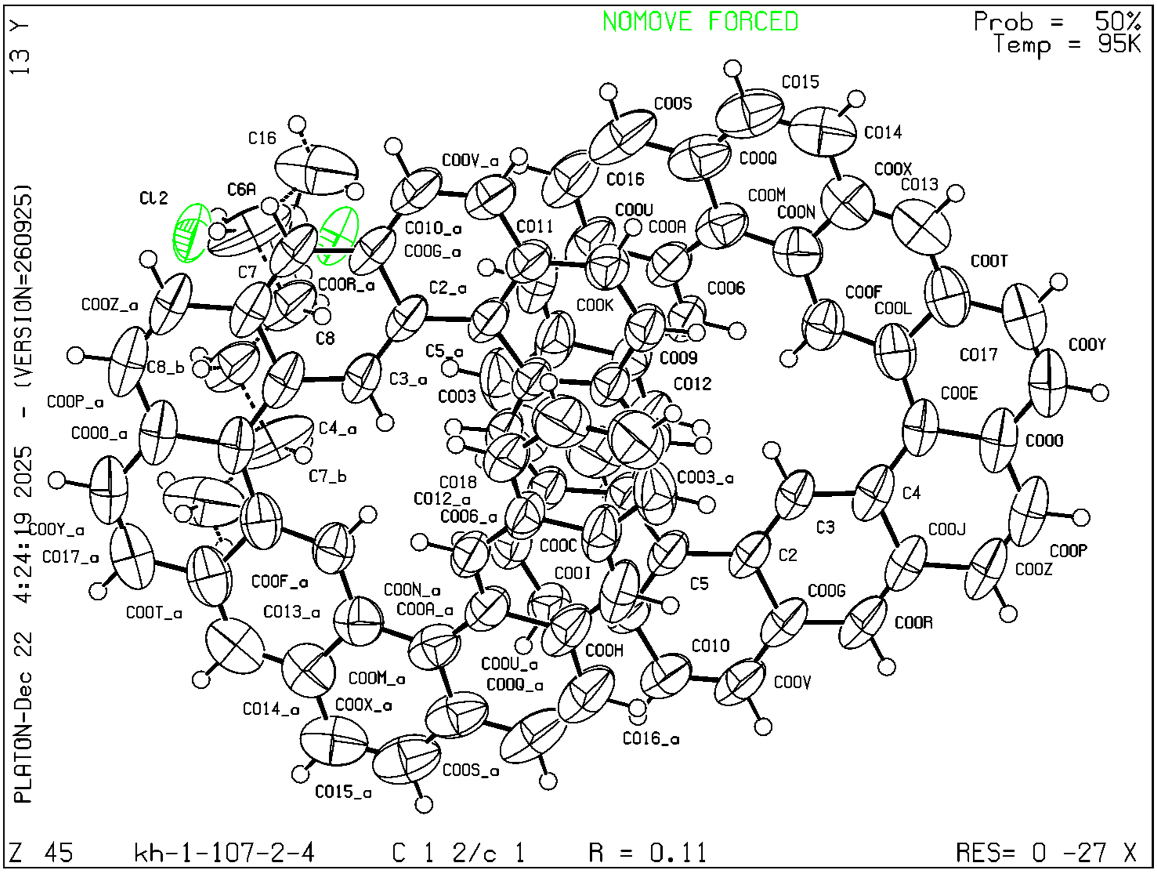

Supplement: Supplementary file 2 [file CHEM-32-e71249-s002.zip › chem71249-sup-0002-cif/KH-1-107-2-4_cifreport_2.pdf]
